# Tailored exercise management versus usual care for people aged 80 years or older with hip/knee osteoarthritis and comorbidities (TEMPO): multicentre feasibility randomised controlled trial in England

**DOI:** 10.1136/bmjopen-2025-104813

**Published:** 2025-09-22

**Authors:** Philippa J A Nicolson, Melanie A Holden, Ioana Marian, Elnaz Saeedi, Esther Williamson, Dianna Moylan, Megan Stone, Sally Hopewell, Sarah E Lamb, TEMPO feasibility trial collaborators, Karen Barker

**Affiliations:** 1 Nuffield Department of Orthopaedics, Rheumatology and Musculoskeletal Sciences (NDORMS), University of Oxford, Oxford, UK; 2 School of Medicine, Keele University, Keele, UK; 3 Oxford Clinical Trials Research Unit, Centre for Statistics in Medicine, Nuffield Department of Orthopaedics, Rheumatology and Musculoskeletal Sciences, University of Oxford, Oxford, UK; 4 Faculty of Health and Life Sciences, University of Exeter, Exeter, UK; 5 Patient Partner, Oxford, UK

**Keywords:** Exercise, Rehabilitation Medicine, Feasibility Studies, Frail Elderly

## Abstract

**Objective:**

To assess the feasibility of conducting a definitive randomised controlled trial (RCT) to test the clinical and cost-effectiveness of a tailored exercise intervention compared with usual care for people aged 80 years and older with hip and/or knee osteoarthritis (OA) and comorbidities.

**Design:**

Two-arm, parallel-design, multicentre, pragmatic, feasibility RCT.

**Setting:**

Four National Health Service outpatient physiotherapy services across England.

**Participants:**

Adults aged 80 years and over with clinical hip and/or knee OA and ≥1 comorbidity.

**Interventions:**

Participants were randomised 1:1 via a central web-based system to be offered: (1) a 12-week tailored exercise programme or (2) usual care. Participants and outcome assessors were not blinded to treatment allocation.

**Feasibility objectives:**

(1) Ability to screen and recruit participants; (2) retention of participants at 14-week follow-up; (3) intervention fidelity (proportion of participants who received ≥4 intervention sessions as per protocol) and (4) participant engagement (assessed by home exercise adherence).

**Results:**

Between 12 May 2022 and 26 January 2023, 133 potential participants were screened, of whom 94 were eligible. The main reasons for ineligibility were symptoms not consistent with hip or knee OA (10/39, 25.6%) or already having had a physiotherapy appointment (8/39, 20.5%). 51 of 94 (54%) eligible participants were recruited. Participants had a mean age of 84 years (SD 3.5), 31 (60.8%) were female and 96.1% reported their ethnicity as White British (n=49/51). 45 of 51 participants (88%) provided outcome data at the 14-week follow-up time point. Four or more intervention sessions were attended by 13/25 (52%) participants. Home exercise log completion declined over time: 6/23 participants (26.1%) returned completed exercise logs for all 12 weeks. The median number of days home exercises were recorded each week was 5 (range 0–7).

**Conclusions:**

This study demonstrated that a definitive trial would be feasible. Before proceeding, modifications to ensure recruitment of a diverse population and intervention fidelity should be addressed.

**Trial registration number:**

ISRCTN75983430.

**STRENGTHS AND LIMITATIONS OF THIS STUDY**

- This study enrolled people aged 80 years and older with hip and/or knee osteoarthritis and comorbidities.
- The study was conducted in four National Health Service outpatient physiotherapy services across England.
- We assessed the feasibility of conducting a large multicentre randomised trial in terms of the ability to screen, recruit and retain participants; intervention fidelity; and participant engagement.
- Participants and outcome assessors were not blinded to treatment allocation.

## Introduction

 Osteoarthritis (OA) of the hip and/or knee is the most common joint disease in the UK, and a leading cause of years lived with disability worldwide.^1 2^ The prevalence of hip and/or knee OA is strongly associated with increasing age.^3^ In 2021, there were 3.2 million people aged 80 years or older in the UK, and this group is projected to exceed 5 million over the next 20 years, making it the fastest-growing population group.^4^ Compared with other adults in England, those aged 80 years or older are the least physically active and have the highest healthcare expenditure.^5^

Hip and/or knee OA has one of the highest rates of comorbidity (68–85%) among all chronic conditions.^6^,^8^ In a UK general practice population of adults aged 50 years and older, people with OA had a 2.35 times higher risk of having comorbidities compared with people without OA.^9^ The presence of comorbidities is also increasingly common with age, meaning people aged 80 years and older with hip and/or knee OA are most likely to also be managing at least one other chronic health condition. The most common conditions among people with hip and/or knee OA include cardiovascular diseases, depression, type 2 diabetes, hypertension and other sites of musculoskeletal pain.^10^,^12^ Our previous research, and that of others, has found that comorbidity increases the likelihood of poor physical function in patients with OA, and the combined influence is greater than would be expected from the influence of either OA or the comorbid conditions alone.^9 13^ Consequently, addressing comorbidities is crucial in optimising the management of OA.^9^

Exercise is recommended by all current evidence-based guidelines for everyone with hip and/or knee OA, regardless of age, pain levels, disease severity or functional ability.^114^,^16^ However, these recommendations are based on data from randomised controlled trials (RCTs) conducted in patients with an average age of 60–65 years.^17 18^ Of the 96 RCTs included in the recently published Cochrane review of exercise for knee OA,^19^ only one trial recruited participants with an average age over 80 years.^20^ Our systematic review of the effects of therapeutic exercise on physical and psychosocial outcomes in any community-dwelling adults aged 80 years or older identified only 16 published RCTs.^21^ People aged 80 and over have been deemed ‘the great forgotten’ in clinical studies,^22^ and the National Institute for Health and Care Research (NIHR) has identified people aged 75 and over as an underserved group in clinical research.^23^

In clinical practice, almost half of people aged 75 years and over in the UK consult their General Practitioner (GP) about managing OA symptoms.^24^ However, research has demonstrated that these patients are significantly less likely to be referred for exercise than younger people.^25^ These low referral rates may reflect uncertainty around exercise prescription for this older patient group, particularly in the presence of comorbidities,^26^ lack of existing suitable exercise interventions or reluctance from patients to engage with a referral for therapeutic exercise.^27^

### Objectives

The aim of the Tailored Exercise Management for People aged 80 years or older with hip/knee Osteoarthritis (TEMPO) study was to assess the feasibility of conducting a definitive RCT to compare the clinical and cost-effectiveness of a tailored exercise intervention to usual care in adults aged 80 years and over with hip and/or knee OA and comorbidities.

The primary feasibility objectives of the trial were to:

- Assess participant recruitment to the trial, measured by the recruitment and consent rates of those eligible.
- Determine participant retention in the trial, measured by the proportion of participants providing outcome data at 14 weeks.

Secondary feasibility objectives were to:

- Assess intervention fidelity by the number, content and timeframe of therapy sessions provided.
- Assess participant engagement by home exercise adherence.
- Assess the content of the usual care intervention.

## Methods

### Study design

A multicentre, parallel, two-group RCT. Participants were allocated to either (1) usual care or (2) a 12-week tailored exercise programme (4–8 sessions of individual physiotherapy). Ethical approval was gained from the London—Brent Research Ethics Committee Reference: 21/LO/0777, and the study was prospectively registered (ISRCTN75983430). The feasibility trial protocol has been published.^28^ An embedded qualitative study including both patient participants and physiotherapists who delivered the intervention was also completed as part of a process evaluation, which will be reported separately.

### Setting

Potentially eligible participants were identified from referrals to four participating National Health Service (NHS) physiotherapy outpatient services, via screening of general practice records and identification of eligible individuals from a cohort study (Oxford Pain, Activity and Lifestyle (OPAL)) run by our research group.^29^ The OPAL study is a prospective longitudinal cohort study of community-dwelling older adults aged 65 years or older. Potentially eligible participants were given study information and, if interested, were invited to screening, first via a telephone call and second in a research clinic assessment at the local site. The study intervention was delivered within physiotherapy outpatient services at four NHS sites.

### Study participants

Full inclusion and exclusion criteria are detailed in online supplemental table S1. The target population was adults aged 80 years or older with clinical hip and/or knee OA (hereafter referred to as hip/knee OA) and one or more comorbidities.

Participants were excluded if they had a terminal illness, significant cognitive impairment, were unable to walk 3 m with or without an aid or were unable to follow verbal or written instructions.

### Randomisation

Consented participants were randomised 1:1 to the tailored exercise programme or usual care using the centralised web-based randomisation service provided by the Oxford Clinical Trials Research Unit. The site’s research clinician undertook randomisation directly themselves. Randomisation was computer-generated and stratified by centre.

### Blinding

Physiotherapists delivering the tailored exercise programme and study participants were told the treatment allocation. The local researcher clinicians conducting the baseline and 14-week assessments were independent of the clinical team who delivered the tailored exercise programme and were not blinded to treatment allocation. The trial statistician was not blinded to treatment allocation as they were responsible for conducting randomisation checks.

### Interventions

The interventions are outlined in online supplemental table S2 based on the Template for Intervention Description and Replication (TIDieR) guidance.^30^ These are summarised below.

#### Tailored exercise programme

Participants in the tailored exercise intervention were offered a minimum of 4 and a maximum of 8 one-to-one sessions with a physiotherapist over 12 weeks. The first session was 60 min in duration, and all other sessions were 30 min. Sessions 1–4 were conducted face-to-face. Sessions 5–8 were at the discretion of the physiotherapist and the participant and could be delivered in-person face-to-face, via video consultation or telephone call.

The tailored exercise programme has been previously described in detail.^31^ In the first session, participants were given a workbook which included information about exercise for hip/knee OA, the benefits of exercise for common comorbidities and safety when exercising. Participants set a functional goal and completed a home exercise action plan. The workbook also included exercise demonstration photos and instructions, prompt cards to remind participants to complete their home exercises and an exercise diary, which they filled out and returned at each session.

The exercise programme consisted of aerobic, joint mobility, lower limb strengthening and balance exercises, targeted to functional activities such as getting out of a chair, walking and climbing stairs. The physiotherapist and the participant selected which exercises were most appropriate based on the assessment and the participant’s functional goals. Each exercise was tailored based on the participant’s physical capability and the presence of comorbidities. Exercise adaptations for common comorbidities were supplied to treating physiotherapists. Up to four of the exercises carried out during the physiotherapy sessions were continued at home, and participants were asked to complete the exercises or home walk on 3 or more days each week.

If the physiotherapist and participant agreed that supervised walking should be a focus, this was included in each session, using additions or obstacles as appropriate to challenge balance.

#### Comparator: usual care

Participants in the usual care intervention were offered an educational booklet^32 33^ at the conclusion of their baseline research clinic assessment. Participants randomised to the usual care intervention who were identified via existing referrals to NHS physiotherapy outpatient departments were offered treatment as usual by a physiotherapist who had not been trained in the TEMPO intervention. Participants randomised to usual care who were identified by screening of GP records, or screening of participants in the OPAL cohort study, continued to be managed by their GP.

### Concomitant care

Other aspects of health and social care continued as normal. Details of additional treatments, including contact with their GP or other health professionals, were recorded at the 14-week assessment.

### Training and monitoring of intervention delivery

Physiotherapists delivering the tailored exercise programme attended a 3-hour training session and were provided with written materials outlining the programme content and practical delivery of the intervention. Intervention provision was recorded by physiotherapists in treatment logs for each contact with participants.

Usual care content was recorded on a treatment log by the research clinician at the 14-week assessment, using data from the participant’s electronic medical record.

### Outcome measures

#### Feasibility success criteria

The main aim of the RCT was to determine the feasibility of a future definitive trial. The main uncertainty was whether enough participants would be able to be recruited and retained in the study until the 14-week follow-up. To determine the feasibility of a definitive RCT, the prespecified success criteria were:

- At least 50 eligible participants are identified and agree to take part over 9 months.
- At least 40% of eligible potential participants consent to be randomised.
- At least 85% of participants provide outcome data at 14 weeks.
- At least 75% of participants receive the allocated intervention sessions as per protocol (minimum of 4 sessions delivered over 12 weeks).

Progression criteria to assess the feasibility of a future definitive trial were assessed using a traffic light system for quantitative feasibility outcomes.^34^ ‘Green’ indicates feasible with current procedures, ‘Amber’ indicates modification to one or more components of the protocol is required to proceed and ‘Red’ indicates a definitive trial would not be considered feasible (table 1).

**Table 1:** Feasibility progression criteria traffic-light summary table

Criteria	Green (go)	Amber (amend)	Red (stop)	Outcome
Recruitment	At least 50 eligible participants are identified and agree to take part over 9 months.	Between 35 and 49 eligible participants are identified and agree to participate over 9 months.	<35 eligible participants are identified and agree to participate over 9 months.	51 eligible participants recruited over 8 months.
Consent	At least 40% of eligible potential participants consent to be randomised.	20–39% of eligible potential participants consent to be randomised.	<20% of eligible potential participants consent to be randomised.	54% (51/94) of eligible potential participants consented to be randomised.
Proportion of randomised participants providing 14-week outcome data	At least 85% of participants provide outcome data at 14 weeks.	60–84% of participants provide outcome data at 14 weeks.	<60% of participants provide outcome data at 14 weeks.	88% (45/51) of randomised participants provided outcome data at 14 weeks.
Intervention fidelity	At least 75% of participants receive the allocated intervention sessions as per protocol (a minimum of 4 sessions delivered over 12 weeks).	50–74% of participants receive the allocated intervention sessions as per protocol (a minimum of 4 sessions delivered over 12 weeks).	<50% of participants receive the allocated intervention sessions as per protocol (a minimum of 4 sessions delivered over 12 weeks).	52% (13/25) of participants randomised to the TEMPO intervention received ≥4 sessions within 12 weeks.

TEMPO, Tailored Exercise Management for People aged 80 years or older with hip/knee Osteoarthritis.

#### Clinical outcomes

Participants were invited to attend a research clinic assessment at baseline and 14 weeks after randomisation. If they were unable to attend the 14-week clinic assessment, they were offered a telephone or postal follow-up to complete the self-reported questionnaires.

Mobility and balance were assessed using the Short Physical Performance Battery (SPPB).^35 36^ The test involves brief tests of sit-to-stand from a chair, balance and walking speed.

Patient-reported outcomes were:

- Ability to perform activities of daily life: Nottingham Extended Activities of Daily Life Scale (NEADL).^37^ Scoring ranges from 0 to 22; a higher score indicates better self-reported function. The NEADL was the proposed primary outcome for a definitive trial.
- Health-related quality of life: EuroQol Group 5-Dimension Questionnaire (EQ-5D-5L).^38 39^ The EQ-5D health status scale is mapped onto the EQ-5D-3L valuation set using the Crosswalk Index Value Calculator.^39^ The scale from this set ranges from −0.594, indicating the worst possible health state, to 1.0 and is anchored at 0 (death) and 1.0 (full health). A respondent’s EQ-Visual Analogue Scale gives self-rated health on a scale where the endpoints are labelled from ‘0—worst imaginable health state’ to ‘100—best imaginable health state’.^38^
- Hip and/or knee joint pain: average, worst and walking pain rated on an 11-point Numeric Rating Scale (0–10 scale, higher scores indicate more pain).
- Depressive symptoms: Geriatric Depression Scale (GDS-15)^40^ (possible scores: 0–15). A higher score indicates more depressive symptoms. A score >5 indicates depression.
- Anxiety symptoms: Geriatric Anxiety Scale (GAS10)^41 42^ (possible scores: 0–30). A higher score indicates more anxiety symptoms. A score of ≥12 indicates severe anxiety.
- Falls: Prevention of Falls Network Europe self-report of falls and fall-related injuries in the past 12 months (baseline assessment) and since baseline (14-week assessment).^43^
- Walking aid use (indoors and outdoors).
- Walking confidence: single item from Modified Gait Self-Efficacy Scale: confidence to walk ½ mile (possible scores 1–10). A higher score indicates more confidence.

### Adverse events

Given the age range of the participant population and the nature of exercise interventions, delayed onset of muscle soreness lasting up to 3 days was not recorded as an adverse event. Pain increases of more than 3 days, any exacerbation of other medical conditions or any falls were recorded by treating physiotherapists in the intervention log, or in patient questionnaires at follow-up.

### Sample size

The main feasibility objective, and the basis of the sample size estimate, was participant recruitment per centre. The target sample size was a minimum of 50 participants in at least four sites over a maximum of 9 months. The sample size was based on a target of recruiting 1.5 participants per month per site, assuming that sites would have staggered openings. The sample size was considered sufficient to answer our feasibility objectives and assess the a priori progression criteria based on Teare *et al* recommendations.^44^

### Statistical analysis

Feasibility objectives and clinical outcomes were reported using descriptive statistics. Mean and SD or median and IQR were used for continuous variables and counts, and numbers and percentages were used for any binary or categorical variables. Feasibility objectives were compared with the progression criteria targets. Outcome data were analysed on an Intention-To-Treat basis, with all participants analysed as per their treatment group allocation and differences in outcomes between treatment groups were reported with 95% CI. Analysis of clinical outcomes was performed using linear regression for continuous outcomes adjusted for the number of comorbidities, baseline outcome value and random effect centre. The study was not powered for formal hypothesis testing between the treatment arms; therefore, no p values are provided.

The study is reported using the Consolidated Standards of Reporting Trials guidelines for pilot and feasibility trials.^45^ All analyses were carried out using a centrally validated software, Stata V.16.0.^46^

### Patient and public involvement

Our initial funding application, intervention development, clinical outcome selection, study material development, interpretation and dissemination of study findings were supported by patient and public involvement. Patient and public involvement based on the Guidance for Reporting Involvement of Patients and the Public (GRIPP2) guidance is detailed in online supplemental table S3.^47^

## Results

### Screening, recruitment and baseline characteristics

Participant screening and recruitment commenced on 12 May 2022 and finished on 26 January 2023 when the target sample size was reached. Baseline characteristics of randomised participants are summarised in table 2. Participants had a mean age of 84 (SD 3.5) years, and 31 (60.8%) were female. In total, 96.1% of participants reported their ethnicity as White British (49/51). Half of the participants lived alone (27/51, 52.9%). The most commonly reported comorbidities were other site/s of musculoskeletal pain (single additional site (13/51, 25.5%), multiple additional sites (32/51, 62.7%)), high blood pressure (31/51, 60.8%), hearing problems (25/51, 49.0%) or visual problems (21/51, 41.2%). More than half of participants reported having fallen in the past 12 months (one fall (13/51, 25.5%), more than one fall (12/51, 23.5%)). Three-quarters of participants reported using a walking aid outdoors, either always (23/51, 45.1%) or sometimes (14/51, 27.5%).

**Table 2:** Baseline characteristics summarised by treatment group and overall

	TEMPO (n=25)	Usual care (n=26)	Total (n=51)
Age (years) (mean (SD))	84.4 (3.7)	83.6 (3.3)	84.0 (3.5)
BMI (n (%))			
Underweight (<18.5)	1 (4.0)	0 (0.0)	1 (2.0)
Normal weight (18.5–24.9)	3 (12.0)	5 (19.2)	8 (15.7)
Overweight (25–29.9)	12 (48.0)	11 (42.3)	23 (45.1)
Obese (≥30)	9 (36.0)	10 (38.5)	19 (37.3)
Sex (n (%))			
Male	7 (28.0)	13 (50.0)	20 (39.2)
Female	18 (72.0)	13 (50.0)	31 (60.8)
Ethnicity (n (%))			
White British	24 (96.0)	25 (96.2)	49 (96.1)
White Other	0 (0.0)	1 (3.8)	1 (2.0)
Prefer not to say	1 (4.0)	0 (0.0)	1 (2.0)
Relationship Status (n (%))			
Married/civil union	10 (40.0)	14 (53.8)	24 (47.1)
Unmarried (never married)	1 (4.0)	1 (3.8)	2 (3.9)
Separated/divorced	2 (8.0)	2 (7.7)	4 (7.8)
Widow/widower	12 (48.0)	9 (34.6)	21 (41.2)
Living alone (n (%))			
No	10 (40.0)	14 (53.8)	24 (47.1)
Yes	15 (60.0)	12 (46.2)	27 (52.9)
Current smoking behaviour (n (%))			
Never smoked	8 (32.0)	12 (46.2)	20 (39.2)
Former smoker	15 (60.0)	14 (53.8)	29 (56.9)
Current smoker	2 (8.0)	0 (0.0)	2 (3.9)
Alcohol use in the past 12 months (n (%))			
Twice a day or more	0 (0.0)	1 (3.8)	1 (2.0)
Daily or almost daily	6 (24.0)	3 (11.5)	9 (17.6)
Once or twice a week	9 (36.0)	10 (38.5)	19 (37.3)
Once or twice a month	3 (12.0)	4 (15.4)	7 (13.7)
Special occasions only	3 (12.0)	4 (15.4)	7 (13.7)
Not at all	4 (16.0)	4 (15.4)	8 (15.7)
Current work status (n (%))			
Retired	24 (96.0)	24 (92.3)	48 (94.1)
Semiretired	0 (0.0)	2 (7.7)	2 (3.9)
Looking after home or family	1 (4.0)	0 (0.0)	1 (2.0)
Painful joints (n (%))			
Left hip	10 (40.0)	10 (38.5)	20 (39.2)
Right hip	10 (40.0)	10 (38.5)	20 (39.2)
Both hips	4 (16.0)	3 (11.5)	7 (13.7)
Left knee	16 (64.0)	14 (53.8)	30 (58.8)
Right knee	16 (64.0)	16 (61.5)	32 (62.7)
Both knees	10 (40.0)	9 (34.6)	19 (37.2)
Joints causing most pain (n (%))			
Left hip	5 (20.0)	6 (23.1)	11 (21.6)
Right hip	5 (20.0)	4 (15.4)	9 (17.6)
Left knee	6 (24.0)	6 (23.1)	12 (23.5)
Right knee	9 (36.0)	10 (38.5)	19 (37.3)
Pain felt over the past week (11-point NRS) (mean (SD))			
Average pain	6.1 (1.9)	6.2 (1.8)	6.2 (1.9)
Worst pain	7.2 (2.3)	7.3 (1.7)	7.3 (2.0)
Average pain while walking	6.0 (2.3)	5.8 (2.6)	5.9 (2.5)
Nordic pain questionnaire (n (%))			
No additional hip and knee pain	2 (8.0)	4 (15.4)	6 (11.8)
Single-site additional pain	5 (20.0)	8 (30.8)	13 (25.5)
Multisite pain	18 (72.0)	14 (53.8)	32 (62.7)
Health conditions (n (%))			
Angina or heart troubles	8 (32.0)	9 (34.6)	17 (33.3)
Anxiety	2 (8.0)	4 (15.4	6 (11.8)
Bladder problems	7 (28.0)	10 (38.5)	17 (33.3)
Cancer	6 (24.0)	6 (23.1)	12 (23.5)
Chronic lung disease	3 (12.0)	3 (11.5)	6 (11.8)
Depression	5 (20.0)	6 (23.1)	11 (21.6)
Diabetes	4 (16.0)	3 (11.5)	7 (13.7)
Digestive problems	4 (16.0)	7 (26.9)	11 (21.6)
Hearing problems	11 (44.0)	14 (53.8)	25 (49.0)
High blood pressure	16 (64.0)	15 (57.7)	31 (60.8)
Memory problems	4 (16.0)	4 (15.4)	8 (15.7)
Osteoporosis	7 (28.0)	6 (23.1)	13 (25.5)
Peripheral vascular disease	2 (8.0)	2 (7.7)	4 (7.8)
Stroke	3 (12.0)	4 (15.4)	7 (13.7)
Visual problems	8 (32.0)	13 (50.0)	21 (41.2)
NEADL (mean (SD))	19.2 (3.0)	19.0 (3.3)	19.1 (3.1)
EQ-5D-5L (mean (SD))			
Utility	0.57 (0.23)	0.62 (0.17)	0.60 (0.20)
VAS	63.4 (20.3)	69.4 (13.4)	66.5 (17.2)
GDS-15 (mean (SD))	3.5 (2.8)	3.0 (2.3)	3.3 (2.6)
GAS10 (mean (SD))	4.8 (4.4)	4.0 (2.6)	4.4 (3.6)
SPPB (mean (SD))	7.1 (3.0)	8.0 (2.8)	7.5 (2.9)
Single item from Modified Gait Self-Efficacy Scale—confidence to walk ½ mile (mean (SD))	5.1 (3.9)	6.5 (3.5)	5.8 (3.7)
Walking aid used to walk outside (n (%))			
No	7 (28.0)	7 (26.9)	14 (27.5)
Yes	12 (48.0)	11 (42.3)	23 (45.1)
Sometimes	6 (24.0)	8 (30.8)	14 (27.5)
Falls in the past 12 months (n (%))			
I have not fallen in the last year	12 (48.0)	14 (53.8)	26 (51.0)
I have fallen once in the last year	8 (32.0)	5 (19.2)	13 (25.5)
I have fallen more than once in the last year	5 (20.0)	7 (26.9)	12 (23.5)
Have you had any broken bones in the last 12 months as a result of falling (n (%))			
No*	13 (100.0)	12 (100)	25 (100.0)
Paid or unpaid carer (n (%))			
No	17 (68.0)	18 (69.2)	35 (68.6)
Yes	8 (32.0)	7 (26.9)	15 (29.4)
Site (n (%))			
Birmingham	8 (32.0)	8 (30.8)	16 (31.4)
Mersey Care	4 (16.00	4 (15.4)	8 (15.7)
Oxford	5 (20.0)	6 (23.1)	11 (21.6)
Sheffield	8 (32.0)	8 (30.8)	16 (31.4)

NEADL: scores 0–22. Higher score = better self-reported function.

GDS-15: possible scores: 0–15. Higher score = more depressive symptoms. >5 = indicates depression.

GAS10: possible scores: 0–30. Higher score = more anxiety symptoms. 12 or more = severe anxiety.

SPPB: possible scores: 0–12. Higher score = better physical performance.

Self-efficacy walking: possible score 1 (no confidence) to 10 (complete confidence).

* All reporting one or more falls in the last 12 months confirmed no broken bones.

BMI, body mass index; EQ-5D-5L, EuroQol Group 5-Dimension Questionnaire; GAS10, Geriatric Anxiety Scale; GDS-15, Geriatric Depression Scale; NEADL, Nottingham Extended Activities of Daily Life Scale; NRS, Numerical Rating Scale; SPPB, Short Physical Performance Battery; TEMPO, Tailored Exercise Management for People aged 80 years or older with hip/knee Osteoarthritis; VAS, Visual Analogue Scale.

### Feasibility objectives

#### Participant recruitment

**Figure 1:**
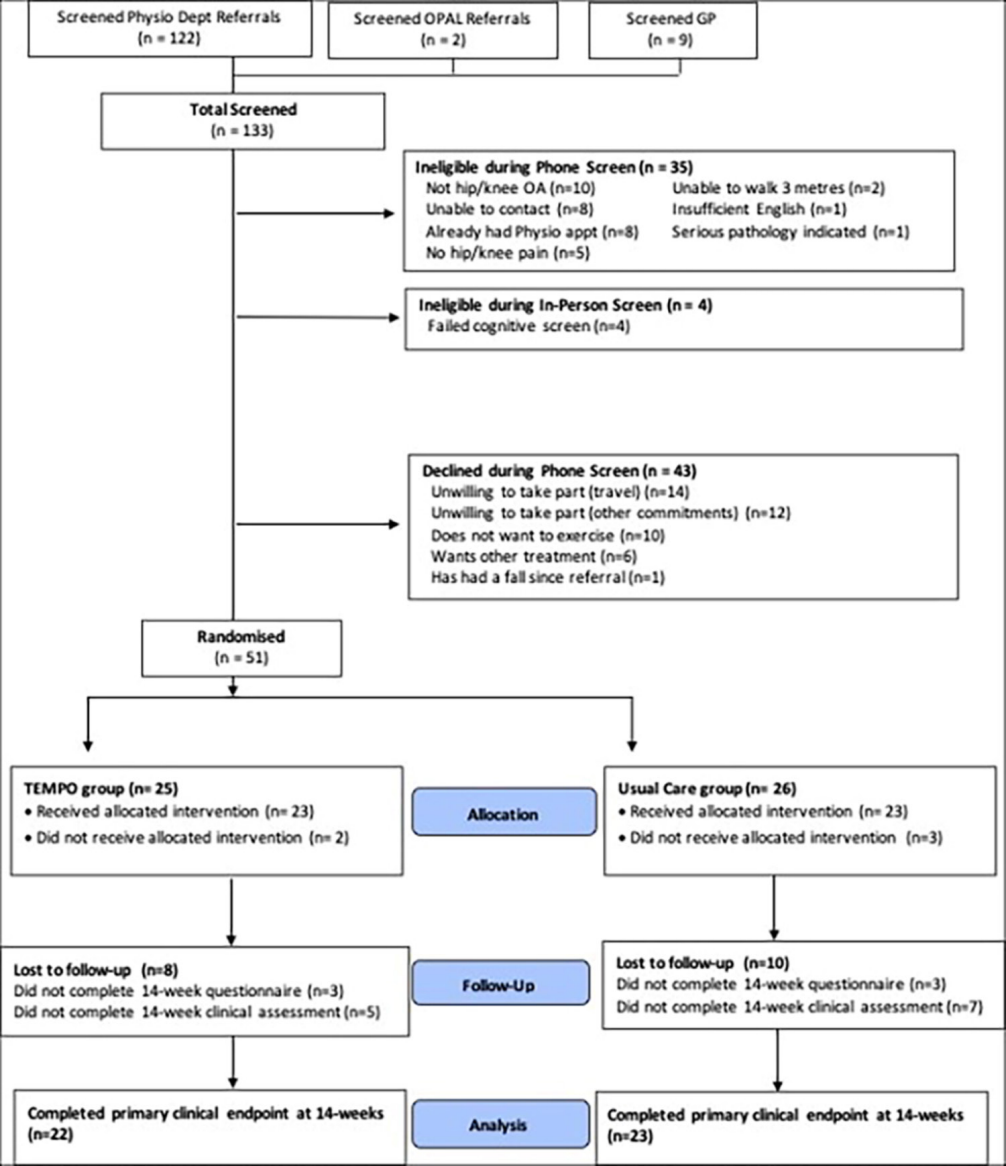
Flow of participants through the tailored exercise management versus usual care for people aged 80 years or older with hip/knee OA and comorbidities (TEMPO) trial. GP, General Practitioner; OA, osteoarthritis; OPAL, Oxford Pain, Activity and Lifestyle; TEMPO, Tailored Exercise Management for People aged 80 years or older with hip/knee Osteoarthritis.

Of the 133 potential participants screened, 94 were eligible, and 51 were recruited and randomised (see figure 1). The most common reason that potential participants were deemed ineligible during phone screening was that their symptoms were not consistent with hip and/or knee OA (10/35, 29%). Potential participants who were deemed ineligible during in-person screening all failed the cognitive screen (4/55, 7%). Of the 43 potential participants who were eligible but declined participation during phone screening, the main reason provided was travel difficulties to and from the study site for assessment and treatment sessions (18/43, 42%). No potential participants declined participation at the in-person screening. The overall recruitment rate was 1.5 participants per month per site. Recruitment (51 participants recruited over 8 months) and consent (54% of eligible potential participants consented to be randomised) exceeded the ‘green’ criteria of 50 participants recruited in 9 months and 40% of eligible participants consenting to be randomised, respectively.

#### Participant retention

At 14-week follow-up, 45/51 participants (88.2%) remained in the trial and provided self-reported questionnaire data. Two participants withdrew over the course of the trial; one because they had other health concerns they needed to focus on, and the other because they were receiving physiotherapy treatment at another hospital. Retention was consistent across allocated groups (22/25, 88.0% randomised to the TEMPO intervention, 23/26, 88.5% randomised to usual care). Completion rate for the physical performance assessment in the clinic was 76.5% (39/51). Participant retention (88% provided 14-week outcome data) exceeded the ‘green’ feasibility progression criteria (85% provided 14-week outcome data).

#### Intervention fidelity

Of the 25 participants randomised to the TEMPO intervention, 23/25 (92.0%) received at least 1 intervention session, and 13/25 (52.0%) received 4 or more sessions as per the protocol (see table 3). When compared with those who attended 4 or more sessions, participants who attended less than 4 sessions were more likely to be male (42% vs 15%), less likely to live alone (50% vs 69%) or use a walking aid inside (25% vs 46%) and reported being more confident walking ½ mile (median score: 8.5/10 vs 3.0). The median number of days from randomisation to the first intervention session was 28 (ie, 4 weeks), and the range was 8–74 days. 20 participants (80%) received all their TEMPO intervention sessions within the goal timeframe of 12 weeks. The most common reasons for attendance at less than 4 sessions were cancelled appointment and unable to rebook 4/10 (40.0%), and discharged from TEMPO by treating physiotherapist 3/10 (30.0%). Intervention fidelity (52% of participants randomised to the TEMPO intervention received ≥4 sessions) met the ‘amber’ feasibility progression criteria (50%–74%), indicating modification to one or more components of the protocol is required to proceed.

**Table 3:** TEMPO intervention fidelity

Treatment received	TEMPO intervention	
	**n=25**	**%**
Treatment attendance		
Not attended any sessions	2	8
Attended less than 4 sessions	10	40
Attended 4 or more sessions	13	52
Sessions attended, median (IQR)	4 (3–5) (Range: 0–8)	
Number of days from randomisation to the first session, median (IQR), (range)	23	28 (13–35) (Range: 8–74)
Number of days from the first session to the last session, median (IQR), (range)§	23	53 (28–70) (Range: 0–111)
Reason for session non-attendance in less than 4 sessions (n=10)		
Cancelled appointment and did not rebook (4 sessions)	4	40
Died	1	10
Withdrew before attending first TEMPO session (2 sessions)	2	20
Discharged from TEMPO by physiotherapist (3 sessions)	3	30
Additional reasons for session non-attendance where at least 4 sessions but not all 8 sessions were attended (n=18)		
Participant forgot (3 sessions)	3	12
Participant unwell (8 sessions)	6	24
Transport issues (1 session)	1	4
No longer wishes to take part (2 sessions)	2	8
Other* (4 sessions)	3	12
Intervention details		
TEMPO workbook given	23	92
Missing	2	8
Goals set		
Yes	22	88
No†	1	4
Missing	2	8
Exercise action plan introduced		
Yes	22	88
No‡	1	4
Missing	2	8
Barriers to exercise discussed in session 1		
Yes	22	88
Missing	3	12

* Mixed up appointment time.

† Not set reason: patient unsure of goals today.

‡ Reason: patient had a recent fall and has rib fractures, so not suitable to exercise.

§ There were 20 patients whose sessions were delivered within 12 weeks (84 days).

TEMPO, Tailored Exercise Management for People aged 80 years or older with hip/knee Osteoarthritis.

Levels of fidelity in delivery of the TEMPO intervention components were variable. All participants who attended one or more TEMPO intervention sessions were prescribed exercises and given the TEMPO workbook (23/23, 100%). Goals were set, an exercise action plan was introduced and barriers to exercise were discussed with 22/23 (96.5%) participants who attended one or more TEMPO sessions.

Comorbidity exercise adaptations were not commonly used in TEMPO intervention sessions. When adaptations were included, they were most often a change of exercise location from curtained cubicles to private rooms due to hearing difficulties (4/23, 17%); balance focus (1/23, 4%); use of blood pressure monitor (2/23, 9%) or relaxation breathing exercises (2/23, 9%). The five most common exercises prescribed to be completed at home were: knee extension (69.1%), marching (67.4%), sit-to-stand (63.1%), deep breathing (58.3%) and trunk rotations (43.8%) (online supplemental table S4). Use of non-protocol treatments was not reported in any TEMPO intervention sessions.

#### Home exercise adherence

Among the 23 participants randomised to the TEMPO intervention who attended at least 1 session, home exercise adherence logs were returned for one or more weeks by 18 participants (78.3%). Home exercise adherence log completion declined over the 12 weeks of the intervention: 6/23 participants (26.1%) returned completed exercise logs for all 12 weeks (online supplemental table S5). The median number of days home exercises were recorded each week over the 12-week intervention was 5 (range 0–7). The median number of days each week that a walk was recorded over the 12-week intervention was also 5 (range 0–7).

Participants randomised to usual care who were referred for physiotherapy as part of their care received a median of 2 physiotherapy sessions (range 0–4 sessions). Most usual care physiotherapy sessions included provision of a home exercise programme (22/26, 85%) and exercises performed in the clinic (18/26, 69%). Less than a quarter of participants who received physiotherapy in the usual care arm were given education or information beyond the booklet given at the trial baseline assessment (6/26, 23%) (online supplemental table S6).

A summary of the feasibility progression criteria traffic light assessment is presented in table 1.

### Outcome measure data collection

Data for patient-reported and clinical assessed outcomes at baseline and 14-week follow-up are summarised in table 4. The proposed primary outcome for a definitive trial was the NEADL. No significant changes were observed from baseline to follow-up assessment in the NEADL, EQ-5D, GDS-15 or GAS10 for either participant group. Data for the NEADL were significantly skewed at both timepoints towards higher self-reported function, and a ceiling effect was noted (median baseline score for both groups: 20.0 (IQR 17.0 to 22.0). Scores for the SPPB increased in both groups, indicating small improvements in physical function. Walking confidence increased in the TEMPO intervention group (baseline median: 5.0; 14-week median: 6.5) and decreased in the usual care group (baseline median: 6.5; 14-week median: 6.0).

**Table 4:** Clinical outcomes at baseline and 14 weeks follow-up

	Time point	TEMPO intervention		Median (IQR)	n	Usual care		Between-group difference
		N	Mean (SD)			Mean (SD)	Median (IQR)	(95% CI)*
NEADL	Baseline	25	19.2 (3.0)	20.0 (17.0 to 22.0)	26	19.0 (3.3)	20.0 (17.0 to 22.0)	
	14 Weeks	22	17.9 (3.7)	19.0 (16.0 to 20.0)	23	17.9 (4.9)	19.0 (15.0 to 21.0)	0.14 (−1.94 to 2.21)
EQ-5D utility score	Baseline	25	0.57 (0.23)	0.59 (0.52 to 0.69)	26	0.62 (0.17)	0.63 (0.58 to 0.72)	
	14 Weeks	22	0.57 (0.28)	0.62 (0.48 to 0.75)	23	0.64 (0.16)	0.69 (0.54 to 0.75)	0.04 (−0.04 to 0.13)
EQ VAS	Baseline	25	63.4 (20.3)	70.0 (50.0 to 80.0)	26	69.4 (13.4)	72.5 (60.0 to 75.0)	
	14 Weeks	22	69.9 (16.1)	70.0 (60.0 to 80.0)	23	71.7 (13.3)	70.0 (60.0 to 80.0)	−0.63 (−7.50 to 6.23)
GDS-15	Baseline	25	3.5 (2.8)	3.0 (1.0 to 5.0)	26	3.0 (2.3)	3.0 (1.0 to 4.0)	
	14 Weeks	22	4.1 (3.4)	3.0 (2.0 to 6.0)	22	3.5 (3.2)	3.0 (1.0 to 4.0)	−0.39 (−2.16 to 1.36)
GAS10	Baseline	25	4.8 (4.4)	4.0 (2.0 to 6.0)	26	4.0 (2.6)	4.0 (2.0 to 5.0)	
	14 Weeks	22	5.4 (5.7)	3.5 (1.0 to 7.0)	23	5.2 (3.4)	4.0 (3.0 to 8.0)	0.25 (−1.88 to 2.38)
SPPB	Baseline	25	7.1 (3.0)	7.0 (5.0 to 10.0)	26	8.0 (2.8)	8.0 (6.0 to 10.0)	
	14 Weeks	19	7.7 (3.4)	8.0 (6.0 to 11.0)	20	8.8 (2.6)	9.0 (7.0 to 11.0)	0.10 (−1.03 to 1.22)
Pain felt over the past week (11-point NRS)								
Average pain	Baseline	25	6.1 (1.9)	6.0 (5.0 to 7.0)	26	6.2 (1.8)	6.0 (5.0 to 7.0)	
	14 Weeks	22	5.0 (3.1)	5.0 (2.0 to 8.0)	23	5.5 (2.4)	5.0 (4.0 to 8.0)	0.26 (−1.02 to 1.56)
Worst pain	Baseline	25	7.2 (2.3)	8.0 (7.0 to 9.0)	26	7.3 (1.7)	8.0 (6.0 to 9.0)	
	14 Weeks	22	6.9 (2.8)	8.0 (7.0 to 9.0)	23	6.2 (2.6)	7.0 (5.0 to 8.0)	−0.64 (−1.92 to 0.63)
Average pain while walking	Baseline	25	6.0 (2.3)	6.0 (5.0 to 8.0)	26	5.8 (2.6)	6.5 (4.0 to 8.0)	
	14 Weeks	22	5.6 (2.9)	6.0 (3.0 to 8.0)	23	4.8 (2.8)	5.0 (3.0 to 8.0)	−0.62 (−1.93 to 0.69)
Confidence to walk ½ mile	Baseline	25	5.1 (3.9)	5.0 (1.0 to 9.0)	26	6.5 (3.5)	6.5 (4.0 to 10.0)	
	14 Weeks	22	6.0 (3.5)	6.5 (3.0 to 10.0)	23	6.1 (3.4)	6.0 (3.0 to 10.0)	−1.23 (−2.44 to 0.03)

NEADL: scores 0–22. Higher score = better self-reported function.

EQ-5D utility score: possible scores −0.594 (worst possible health) to 1.0 (full health), anchored at 0 (death).

GAS10: possible scores: 0–30. Higher score = more anxiety symptoms. 12 or more = severe anxiety.

GDS-15: possible scores: 0–15. Higher score = more depressive symptoms. >5 = indicates depression.

VAS: possible scores 0–100. Higher score = better health.

SPPB: possible scores: 0–12. Higher score = better physical performance.

NRS: 11-point NRS. Higher score = worse pain.

Confidence to walk ½ mile: possible score 1 (no confidence) to 10 (complete confidence).

* Analysis adjusted for number of comorbidities, baseline and random effect centre.

EQ-5D, EuroQol Group 5-Dimension Questionnaire; GAS10, Geriatric Anxiety Scale; GDS-15, Geriatric Depression Scale; NEADL, Nottingham Extended Activities of Daily Life Scale; NRS, Numerical Rating Scale; SPPB, Short Physical Performance Battery; VAS, Visual Analogue Scale.

#### Adverse events

No serious adverse events possibly related to the intervention were reported. Unrelated to trial interventions or procedures, one participant died over the course of the trial.

## Discussion

Feasibility criteria related to recruitment, consent rates and participant retention all met the ‘green (go)’ criteria, and intervention fidelity met the ‘amber (amend)’ level. Recruited participants almost all identified as White British, and we observed a mismatch between self-rated and observed physical function among participants.

The study identified several important considerations that should be addressed to optimise the design of a future RCT. Potentially contributing to the limited ethnic diversity of recruited participants is that we recruited only from patients who had already consulted their GP about their OA, and we did not provide an easy-read or translated patient information sheet. Research patient information sheets are well-known to be long, complex and have poor readability. Potential modifications in a future RCT may include alternative methods of recruitment such as advertising in community settings, visiting community groups to speak about the trial and to answer questions, and producing easy-read and/or translated versions of the patient information sheet.

Consideration should also be given to the selection of the primary outcomes for a definitive trial. The NEADL Scale^37^ was selected as it is a global, rather than disease-specific, measure of self-reported function, and was preferred by Patient and Public Involvement (PPI) members. However, we observed a ceiling effect, despite observed physical function scores (measured by the SPPB),^36^ indicating that the majority of participants had moderate mobility limitations. Notably, participants recruited in the trial had comparable comorbidity profiles, observed physical function, self-reported depression, falls and walking confidence scores to those of similar age taking part in other exercise trials,^48^,^50^ but reported higher NEADL scores. Primary outcome selection for a future main trial should ensure that a wide variety of abilities can be assessed, and changes over time can be accurately captured for all participants. Using coprimary outcomes of the SPPB and EQ-5D-5L would enable objective assessment of functional change and broader self-reported assessment of health-related quality of life.

Delivery of the intervention across sites varied, and only half of the participants randomised to the intervention group received 4 or more sessions as per the protocol. Reasons for attendance at less than 4 sessions are commonly related to a lack of capacity in physiotherapists' schedules: being unable to rebook when an appointment was cancelled, or being unable to schedule all sessions within the 12-week intervention period. A future RCT should ensure that clinicians’ time is protected for the delivery of trial intervention sessions or consider extending the intervention period to 16 weeks. Comorbidity adaptations were rarely used by treating clinicians, despite being a key part of the tailored exercise programme. Reasons for this were explored in the embedded qualitative study, which will be reported separately. Future trial clinician training should ensure that clinicians delivering the intervention are confident in all aspects of the programme. The protocol included the option for TEMPO sessions 5–8 to be delivered via telephone or videocall at the discretion of the participant and physiotherapist. There was very little uptake of remote delivery (3 sessions were delivered by telephone across the TEMPO group, and 0 by videocall). Future trial development should give consideration to overcoming barriers to uptake of video consultations in this population group, as they offer an alternative to overcome transport and hospital access difficulties.

We did not define the content of usual care for this feasibility trial. Participants randomised to usual care received a median of 2 physiotherapy sessions and were most commonly prescribed exercises and a home exercise programme. This level of input does not differ significantly from the care received in the intervention arm, and this would need to be considered for a future RCT.

A key strength of the study is that we specifically developed the intervention with, and for, people aged 80 years and older. This population group is under-represented in existing research and is commonly excluded from exercise trials for people with OA. By working with people aged 80 years and older throughout the development phase of the intervention and trial, we were able to incorporate their preferences.^31^ This is reflected in the successful recruitment, consent and retention rates observed. We found that this population was interested in research and willing to participate, particularly when they felt the research was designed for them rather than for a broader adult population. This aligns with the findings of Forsat *et al*^51^ in their systematic review of barriers and solutions for recruitment of older adults to clinical research.

The limitations of the study should be acknowledged when interpreting the findings. Given that 96.1% of all participants identified as White British, the population recruited was not truly representative of the general population aged 80 years and older across the UK, or those with hip/knee OA and comorbidities. Prior to a large RCT, we plan to work in collaboration with community groups to ensure the study is accessible and inclusive for potential participants from a range of ethnic backgrounds.

To our knowledge, this is the first study to assess the feasibility of an RCT to compare the clinical and cost-effectiveness of a tailored exercise intervention to usual care, specifically among adults aged 80 years and over with hip/knee OA and comorbidities. By undertaking a feasibility trial to evaluate key uncertainties, this study has generated important data on the likely success of a future RCT and provided insight into approaches to optimise trial design and processes. We are currently undertaking a process evaluation to more closely examine the uncertainties identified in this feasibility RCT. We are also currently working with communities to identify ways to ensure a future RCT includes diverse ethnic populations.

## Supplementary material

10.1136/bmjopen-2025-104813 online supplemental file 1

10.1136/bmjopen-2025-104813 online supplemental file 2

10.1136/bmjopen-2025-104813 online supplemental file 3

10.1136/bmjopen-2025-104813 online supplemental file 4

10.1136/bmjopen-2025-104813 online supplemental file 5

10.1136/bmjopen-2025-104813 online supplemental file 6

## Data Availability

Data are available upon reasonable request.
