# Supplementary material for: Tailored exercise management versus usual care for people aged 80 years or older with hip/knee osteoarthritis and comorbidities (TEMPO): multicentre feasibility randomised controlled trial in England
Source: BMJ Open. 2025 Sep 22;15(9):e104813. doi: 10.1136/bmjopen-2025-104813 (PMC12458626; doi:10.1136/bmjopen-2025-104813)
Supplement: online supplemental file 1 [file bmjopen-15-9-s001.docx]

**Supplementary Table S1. Inclusion and exclusion criteria**

| **Inclusion criteria** |
| --- |
| Registered with a primary care practice |
| ≥80 years of age |
| Knee and/or hip osteoarthritis. GP register check (hip/knee osteoarthritis diagnosis recorded) or self-report of: knee or hip joint pain lasting 3 months or longer AND knee or hip joint pain on most days of the past month. |
| ≥1 comorbidity. GP register check and/or self-report |
| Individual is willing and able to give informed consent for participation in the study |
| **Exclusion criteria** |
| Has a terminal condition with a life expectancy of less than 6 months or under palliative care |
| Any substantial health or social concern that, in the opinion of the inidividual’s GP, would place the individual at increased risk or inability to participate including known inability to provide informed consent. |
| Significant cognitive impairment. GP check and/or assessed by research clinician. Eligibility screen includes the 6-item Cognitive Impairment Test (score of 8 or more are ineligible). |
| Unable to walk 3 metres with or without an aid |
| Presents with signs of serious pathology requiring immediate referral for investigations |
| Unable to follow verbal or written instructions including inability to follow simple safety instructions |
