# Supplementary material for: Tailored exercise management versus usual care for people aged 80 years or older with hip/knee osteoarthritis and comorbidities (TEMPO): multicentre feasibility randomised controlled trial in England
Source: BMJ Open. 2025 Sep 22;15(9):e104813. doi: 10.1136/bmjopen-2025-104813 (PMC12458626; doi:10.1136/bmjopen-2025-104813)
Supplement: online supplemental file 2 [file bmjopen-15-9-s002.docx]

**Supplementary Table S2. Intervention descriptions based on TIDieR guidance.**

| TIDieR items | Description | |
| --- | --- | --- |
| Brief name | TEMPO (Tailored Exercise Management for People aged 80 years or older with hip/knee Osteoarthritis) | |
| Why | All evidence-based guidelines recommend exercise for everyone with hip/knee osteoarthritis, but people aged 80 years and older are commonly excluded from these trials, and evidence in this population is very limited. | |
|  | Tailored exercise | Usual care |
| What | 4-8 one-to-one sessions with a physiotherapist over 12 weeks: tailored exercise, education, and a home exercise programme. | Usual care as determined by GP. |
| Materials: participants | Participant workbook: education, home exercise instructions and photos, exercise action planner, home exercise prompts, home exercise diary.  Ankle cuff weights (if appropriate) | Versus Arthritis education booklet. |
| Materials: physiotherapists | Training session pack detailing study and intervention procedures.  Quick reference guide detailing exercise adaptions. | No materials supplied. |
| Training | Three-hour face-to-face training delivered by TEMPO trial research physiotherapist.  Training pack detailing all aspects of the trial and the tailored exercise programme. | No training given. |
| Procedures | Appointment 1:  Assess participant as per normal physiotherapy practice.  Issue participant workbook. Discuss content of workbook.  Discuss barriers to exercise.  Help participant to set an outcome goal.  Agree exercises to commence, complete these and agree which to include in home programme.  Mark home exercises in workbook.  Help participant to complete the home exercise action planner.  Make follow-up appointment.  Complete treatment log.  Appointments 2-8:  Reassess as per normal physiotherapy practice.  Discuss barriers to exercise.  Review exercises, progress/regress or change as appropriate. Agree which to include in home programme.  Mark home exercises in workbook.  Make follow-up appointment.  Complete treatment log (after every appointment). | As determined by treating health professional. |
| Who provides | Physiotherapists (Band 6 or above) already working in participating NHS musculoskeletal outpatient departments. | GP. If recruited from existing physiotherapy department referral participant seen as usual by a physiotherapist not trained in the TEMPO intervention. |
| How | Participants receive 4-8 one-to-one sessions with a physiotherapist over 12 weeks.  Appointment 1: 60 minutes. In clinic.  Appointments 2-4: 30 min each. In clinic.  Appointment 5-8: 30 min each. In clinic, via video or via telephone. | As determined by treating health professional. |
| Where | NHS musculoskeletal physiotherapy outpatient departments in the UK.  Home exercise programme performed by the participant at home. | If referred for physiotherapy: NHS musculoskeletal physiotherapy outpatient departments in the UK. |
| When and how much | Appointment 1: within two weeks of baseline assessment.  Appointments 2-8: within 12 weeks. | As determined by treating health professional. |
| Tailoring | Education: Focus of education and advice was individualised based on assessment.  Exercises: Comorbidity adaptions were suggested as detailed in Appendix X.  Exercise selection, number of repetitions, sets and/or load was at the discretion of the treating physiotherapist, considering the participants ability, comorbidities and preferences. | As determined by treating health professional. |
| Modifications |  |  |
| Intervention fidelity | | |
| How well: training | Training delivery, content, duration, and confidence to implement the programme were evaluated via semi-structured qualitative interviews. | N/A |
| How well: physiotherapists | Treatment logs.  Site monitoring visits. | Usual Care log completed by research clinician using electronic patient record at follow-up assessment. |
| How well: participants | Attendance recorded in treatment logs.  Home exercises recorded in home exercise diary. | Not assessed. |
| How well: reporting | Intervention delivery data captured in treatment logs and home exercise diary data was entered onto the trial database and the findings are reported in the results section of this manuscript. | Intervention data captured in Usual Care logs was entered onto the trial database and the findings are reported in the results section of this manuscript. |
| TIDieR: Template for Intervention Description and Replication; GP: General Practitioner; NHS: National Health Service. | | |
