# Supplementary material for: Tailored exercise management versus usual care for people aged 80 years or older with hip/knee osteoarthritis and comorbidities (TEMPO): multicentre feasibility randomised controlled trial in England
Source: BMJ Open. 2025 Sep 22;15(9):e104813. doi: 10.1136/bmjopen-2025-104813 (PMC12458626; doi:10.1136/bmjopen-2025-104813)
Supplement: online supplemental file 3 [file bmjopen-15-9-s003.docx]

**Supplementary Table S3. Patient and Public Involvement summary (GRIPP2 short form checklist)**

| **Aim of PPI** | The aims of PPI were to:   - Guide development of the TEMPO intervention (e.g. delivery options, content, materials). - Provide input to study methods (e.g. recruitment approaches; outcome selection). - Give ongoing input to the running of the trial and dissemination of results. |
| --- | --- |
| **PPI Methods** | Formation of a patient steering group  An eight-member PPI group was established when intervention and trial development work began. Due to COVID-19 restrictions telephone calls were held with members individually on two occasions and members were invited to give feedback on documents via email on two occasions. The selection of clinical outcome measures was guided by the preferences of the PPI group. Feedback from the group, in combination with the qualitative interviews undertaken during the development phase determined the mode of delivery of the intervention, the number of physiotherapy sessions, and content of the TEMPO workbook. A member of the PPI group is also a member of the TMG.  Qualitative interviews with patients during development phase  Qualitative interviews were conducted via telephone (due to COVID-19 restrictions) with 12 patients who would be eligible to participate in the trial, to identify exercise preferences, and barriers and facilitators to exercise participation.  Appointment of patient to trial management group  One member of the patient steering group was invited to join the trial management group. She attended monthly meetings held on Teams throughout the lifecycle of the trial (for which she was reimbursed for her time), and provided input to discussions, problem solving and planning. She reviewed the manuscript and is a co-author. |
| **Results of PPI** | Selection of outcome – PPI group members preferred a ‘broad’ measure of function rather than a disease-specific one. Members also favoured a combination of measuring physical ability and self-reported function.  Intervention – Interviewees and PPI group members had a clear preference for individual sessions, face to face with the clinician. Participants felt that a group setting, at least to begin with, might mean less personalisation of the exercises and multiple participants spoke about feeling uncomfortable being ‘bundled into a group just because we have the same condition or are the same age’. Participants spoke about being self-conscious of their walking ability in public, and of safety concerns about completing home exercises.  Patients identified barriers to participation in the trial, and suggested ways in which these barriers could be overcome (including ensuring that sites were on public transport routes, reimbursing participants for study assessment appointments, and flexible numbers of sessions in the intervention). |
| **Discussion** | PPI had a significant impact on the intervention development and study methods. Forming the patient steering group at the time of working on the funding application worked well as members were familiar with the study and felt that they had the opportunity to really help shape the intervention from the outset.  Intervention development took place during the COVID-19 pandemic, so we were not able to have any face-to-face meetings. There were both positives (patients may have felt more able to be open and honest about their experiences in a one-to-one conversation over the telephone; the potential ‘loudest voice in the room’ effect of a focus group was not an issue) and negatives (group discussion often leads to different issues/suggestions being raised |
| **Reflections** | On reflection it would have been beneficial to have the patient group more involved during the running of the trial. Their input in intervention and trial development was invaluable, but it would be great to keep them involved as a group, updating on progress and discussing issues beyond the single PPI member of the trial management group.  The limitations of undertaking PPI during the COVID-19 pandemic with social restrictions are mentioned above. Ideally, we would have been able to hold group meetings and include both individual and group discussions. |

PPI: Patient and Public Involvement. GRIPP: Guidance for Reporting Involvement of Patients and the Public
