# Supplementary material for: Tailored exercise management versus usual care for people aged 80 years or older with hip/knee osteoarthritis and comorbidities (TEMPO): multicentre feasibility randomised controlled trial in England
Source: BMJ Open. 2025 Sep 22;15(9):e104813. doi: 10.1136/bmjopen-2025-104813 (PMC12458626; doi:10.1136/bmjopen-2025-104813)
Supplement: online supplemental file 4 [file bmjopen-15-9-s004.docx]

**Supplementary Table S4. TEMPO intervention delivery**

| **Session Number**  **Numbers attending (N= )** | **Session 1**  **N = 23** | | **Session 2**  **N = 16** | | **Session 3**  **N = 18** | | **Session 4**  **N = 12** | | **Session 5**  **N = 10** | | **Session 6**  **N = 8** | | **Session 7**  **N = 2** | | **Session 8**  **N = 3** | |
| --- | --- | --- | --- | --- | --- | --- | --- | --- | --- | --- | --- | --- | --- | --- | --- | --- |
| **Mode of delivery** |  |  |  |  |  |  |  |  |  |  |  |  |  |  |  |  |
| In-person | 23 | 100.0% | 16 | 100.0% | 17 | 94.4% | 11 | 91.7% | 10 | 100.0% | 8 | 100.0% | 2 | 100.0% | 2 | 66.7% |
| via Telephone | 0 | 0.0% | 0 | 0.0% | 1 | 5.6% | 1 | 8.3% | 0 | 0.0% | 0 | 0.0% | 0 | 0.0% | 1 | 33.3% |
| via Videocall | 0 | 0.0% | 0 | 0.0% | 0 | 0.0% | 0 | 0.0% | 0 | 0.0% | 0 | 0.0% | 0 | 0.0% | 0 | 0.0% |
| **Session not delivered**  **(denominator is n= 25)** | 2 | 8.0% | 9 | 36.0% | 7 | 28.0% | 13 | 52.0% | 15 | 60.0% | 17 | 68.0% | 23 | 92.0% | 22 | 88.0% |
| **Exercise adaptions** |  |  |  |  |  |  |  |  |  |  |  |  |  |  |  |  |
| **Physiological** |  |  |  |  |  |  |  |  |  |  |  |  |  |  |  |  |
| HR monitor | 1 | 4.3% | 1 | 6.3% | 0 | 0.0% | 0 | 0.0% | 0 | 0.0% | 0 | 0.0% | 0 | 0.0% | 1 | 33.3% |
| BP monitor | 2 | 8.7% | 2 | 12.5% | 2 | 11.1% | 1 | 8.3% | 2 | 20.0% | 1 | 12.5% | 1 | 50.0% | 1 | 33.3% |
| Extended warm-up/Cool-down | 0 | 0.0% | 0 | 0.0% | 0 | 0.0% | 0 | 0.0% | 0 | 0.0% | 0 | 0.0% | 0 | 0.0% | 1 | 33.3% |
| Other | 1 | 4.3% | 0 | 0.0% | 0 | 0.0% | 1 | 8.3% | 0 | 0.0% | 0 | 0.0% | 0 | 0.0% | 0 | 0.0% |
| **Environmental** |  |  |  |  |  |  |  |  |  |  |  |  |  |  |  |  |
| Exercise location | 4 | 17.4% | 1 | 6.3% | 1 | 5.6% | 0 | 0.0% | 0 | 0.0% | 0 | 0.0% | 0 | 0.0% | 0 | 0.0% |
| Instruction modifications | 1 | 4.3% | 0 | 0.0% | 0 | 0.0% | 0 | 0.0% | 0 | 0.0% | 1 | 12.5% | 0 | 0.0% | 0 | 0.0% |
| Balance focus | 1 | 4.3% | 3 | 18.8% | 1 | 5.6% | 2 | 16.7% | 0 | 0.0% | 1 | 12.5% | 1 | 50.0% | 0 | 0.0% |
| Other | 0 | 0.0% | 0 | 0.0% | 0 | 0.0% | 0 | 0.0% | 0 | 0.0% | 0 | 0.0% | 0 | 0.0% | 0 | 0.0% |
| **Behavioural** |  |  |  |  |  |  |  |  |  |  |  |  |  |  |  |  |
| Rescue exercises | 0 | 0.0% | 0 | 0.0% | 0 | 0.0% | 0 | 0.0% | 0 | 0.0% | 0 | 0.0% | 0 | 0.0% | 1 | 33.3% |
| Breathing exercises | 2 | 8.7% | 1 | 6.3% | 1 | 5.6% | 1 | 8.3% | 2 | 20.0% | 2 | 25.0% | 0 | 0.0% | 1 | 33.3% |
| Other | 0 | 0.0% | 0 | 0.0% | 0 | 0.0% | 0 | 0.0% | 1 | 10.0% | 0 | 0.0% | 0 | 0.0% | 0 | 0.0% |
| **Aerobic exercises** |  |  |  |  |  |  |  |  |  |  |  |  |  |  |  |  |
| **Deep breathing** |  |  |  |  |  |  |  |  |  |  |  |  |  |  |  |  |
| Reps* | 16 | 7 (3, 10) | 9 | 7 (5, 10) | 6 | 9 (5, 10) | 4 | 9 (5, 10) | 4 | 9 (5, 10) | 4 | 9 (5, 10) | 1 | 10 (10, 10) | 1 | 5 (5, 5) |
| Sets* | 16 | 1(1,3) | 9 | 1(1,2) | 6 | 2(1,4) | 4 | 1(1,2) | 4 | 1(1,2) | 4 | 2(1,3) | 1 | 1(1,1) | 1 | 2(2,2) |
| Given as home exercise | 12 | 52.2% | 10 | 62.5% | 9 | 50.0% | 8 | 66.7% | 6 | 60.0% | 6 | 75.0% | 2 | 100.0% | 0 | 0.0% |
| **Marching** |  |  |  |  |  |  |  |  |  |  |  |  |  |  |  |  |
| Reps* | 21 | 8(4,10) | 12 | 9(5,20) | 11 | 9(5,10) | 7 | 10(5,15) | 7 | 11(5,20) | 5 | 11(5,20) | - | - | 1 | 10(10,10) |
| Sets* | 21 | 1(1,3) | 12 | 2(1,3) | 11 | 2(1,3) | 7 | 2(1,3) | 7 | 2(1,3) | 5 | 2(1,3) |  |  | 1 | 1(1,1) |
| Given as home exercise | 17 | 73.9% | 13 | 81.3% | 13 | 72.2% | 10 | 83.3% | 7 | 70.0% | 6 | 75.0% | - | - | 1 | 33.3% |
| **Joint mobility exercises** |  |  |  |  |  |  |  |  |  |  |  |  |  |  |  |  |
| **Sitting up – slouching** |  |  |  |  |  |  |  |  |  |  |  |  |  |  |  |  |
| Reps* | 14 | 8(5,10) | 5 | 7(5,10) | 6 | 8(5,10) | 3 | 8(5,10) | 4 | 9(5,10) | 4 | 8(5,10) | 1 | 10(10,10) | 1 | 10(10,10) |
| Sets* | 14 | 1(1,1) | 5 | 1(1,2) | 6 | 1(1,2) | 3 | 1(1,2) | 4 | 1(1,2) | 4 | 1(1,2) | 1 | 1(1,1) | 1 | 1(1,1) |
| Given as home exercise | 10 | 43.5% | 7 | 43.8% | 8 | 44.4% | 7 | 58.3% | 5 | 50.0% | 4 | 50.0% | 1 | 50.0% | 0 | 0.0% |
| **Trunk rotations** |  |  |  |  |  |  |  |  |  |  |  |  |  |  |  |  |
| Reps* | 16 | 8(2,10) | 7 | 8(5,10) | 5 | 7(5,10) | 3 | 8(5,10) | 4 | 9(5,10) | 4 | 9(5,10) | 1 | 10(10,10) | - | - |
| Sets* | 16 | 1(1,3) | 7 | 1(1,3) | 5 | 1(1,2) | 3 | 1(1,2) | 4 | 1(1,2) | 4 | 1(1,2) | 1 | 1(1,1) | - | - |
| Given as home exercise | 11 | 47.8% | 8 | 50.0% | 7 | 38.9% | 6 | 50.0% | 5 | 50.0% | 4 | 50.0% | 2 | 100.0% | 0 | 0.0% |
| **Foot alphabet** |  |  |  |  |  |  |  |  |  |  |  |  |  |  |  |  |
| Reps* | 14 | 10(1,20) | 6 | 8(1,10) | 5 | 11(4,20) | 3 | 13(10,20) | 5 | 16(10,20) | 3 | 17(10,20) | - | - | - | - |
| Sets* | 14 | 1(1,3) | 6 | 1(1,3) | 5 | 1(1,1) | 3 | 1(1,1) | 5 | 1(1,2) | 3 | 1(1,1) | - | - | - | - |
| Given as home exercise | 11 | 47.8% | 6 | 37.5% | 6 | 33.3% | 7 | 58.3% | 6 | 60.0% | 5 | 62.5% | 1 | 50.0% | 0 | 0.0% |
| **Lower limb strengthening exercises** |  |  |  |  |  |  |  |  |  |  |  |  |  |  |  |  |
| **Knee extension** |  |  |  |  |  |  |  |  |  |  |  |  |  |  |  |  |
| Reps* | 16 | 9(5,10) | 12 | 9(5,10) | 11 | 9(5,10) | 6 | 9(5,10) | 6 | 9(5,15) | 4 | 9(5,10) | 2 | 10(10,10) | 2 | 8(5,10) |
| Sets* | 16 | 1(1,3) | 12 | 2(1,3) | 11 | 2(1,3) | 6 | 2(1,3) | 6 | 2(1,3) | 4 | 2(1,2) | 2 | 2(1,3) | 2 | 3(2,3) |
| Weight (kg)* | 16 | 0(0,3) | 12 | 0(0,2.5) | 11 | 1(0,3) | 6 | 1(0,3) | 6 | 1(0,2) | 4 | 1(0,2) | 2 | 3(2,3) | 2 | 2(2,2) |
| Given as home exercise | 12 | 52.2% | 11 | 68.8% | 10 | 55.6% | 9 | 75.0% | 6 | 60.0% | 6 | 75.0% | 2 | 100.0% | 2 | 66.7% |
| **Hip abduction** |  |  |  |  |  |  |  |  |  |  |  |  |  |  |  |  |
| Reps* | 11 | 9(5,10) | 8 | 9(5,10) | 9 | 8(4,10) | 5 | 8(5,10) | 6 | 10(5,15) | 4 | 8(5,10) | - | - | - | - |
| Sets* | 11 | 1(1,3) | 8 | 1(1,3) | 9 | 2(1,3) | 5 | 2(1,2) | 6 | 2(1,3) | 4 | 1(1,2) | - | - | - | - |
| Weight* | 10 | 0(0,0) | 8 | 0(0,1) | 9 | 1(0,2) | 5 | 1(0,3) | 6 | 1(0,2) | 4 | 1(0,3) |  |  |  |  |
| Given as home exercise | 7 | 30.4% | 8 | 50.0% | 8 | 44.4% | 8 | 66.7% | 5 | 50.0% | 4 | 50.0% | 1 | 50.0% | 0 | 0.0% |
| **Hip extension** |  |  |  |  |  |  |  |  |  |  |  |  |  |  |  |  |
| Reps* | 9 | 8(3,10) | 7 | 9(5,10) | 6 | 7(4,10) | 5 | 8(5,10) | 4 | 10(5,15) | 3 | 8(5,10) | - | - | - | - |
| Sets* | 9 | 1(1,1) | 7 | 1(1,2) | 6 | 2(1,3) | 5 | 1(1,2) | 4 | 2(1,3) | 3 | 1(1,2) | - | - | - | - |
| Weight* | 8 | 0(0,0) | 7 | 0(0,1) | 6 | 1(0,2) | 5 | 1(0,3) | 4 | 1(0,2) | 3 | 2(0,3) | - | - | - | - |
| Given as home exercise | 5 | 21.7% | 5 | 31.3% | 5 | 27.8% | 6 | 50.0% | 5 | 50.0% | 4 | 50.0% | 1 | 50.0% | 0 | 0.0% |
| **Sit to stand** |  |  |  |  |  |  |  |  |  |  |  |  |  |  |  |  |
| Reps* | 15 | 7(2,10) | 9 | 9(4,15) | 13 | 9(5,18) | 6 | 7(5,10) | 8 | 9(5,10) | 5 | 11(5,20) | 1 | 5(5,5) | 1 | 2(2,2) |
| Sets* | 15 | 1(1,3) | 9 | 2(1,3) | 13 | 2(1,3) | 6 | 2(1,3) | 8 | 1(1,2) | 5 | 2(1,3) | 1 | 1(1,1) | 1 | 1(1,1) |
| Given as home exercise | 13 | 56.5% | 10 | 62.5% | 13 | 72.2% | 9 | 75.0% | 8 | 80.0% | 6 | 75.0% | 1 | 50.0% | 1 | 33.3% |
| **Squats** |  |  |  |  |  |  |  |  |  |  |  |  |  |  |  |  |
| Reps* | 10 | 8(2,10) | 4 | 10(10,10) | 6 | 10(5,12) | 6 | 8(5,10) | 5 | 9(5,10) | 6 | 8(5,10) | 2 | 8(5,10) | 1 | 5(5,5) |
| Sets* | 10 | 1(1,3) | 4 | 2(1,3) | 6 | 2(1,3) | 6 | 2(1,3) | 5 | 2(1,3) | 6 | 2(1,3) | 2 | 2(1,3) | 1 | 4(4,4) |
| Given as home exercise | 5 | 21.7% | 4 | 25.0% | 7 | 38.9% | 6 | 50.0% | 4 | 40.0% | 4 | 50.0% | 2 | 100.0% | 1 | 33.3% |
| **Step-ups** |  |  |  |  |  |  |  |  |  |  |  |  |  |  |  |  |
| Reps* | 3 | 12(10,15) | 2 | 10(10,10) | 2 | 8(5,10) | 1 | 10(10,10) | 2 | 15(10,20) | 2 | 10(10,10) | 1 | 10(10,10) | 1 | 10(10,10) |
| Sets* | 3 | 1(1,1) | 2 | 1(1,1) | 2 | 2(1,3) | 1 | 1(1,1) | 2 | 2(2,2) | 2 | 3(1,4) | 1 | 3(3,3) | 1 | 3(3,3) |
| Given as home exercise | 2 | 8.7% | 3 | 18.8% | 4 | 22.2% | 2 | 16.7% | 3 | 30.0% | 3 | 37.5% | 2 | 100.0% | 0 | 0.0% |
| **Balance exercises** |  |  |  |  |  |  |  |  |  |  |  |  |  |  |  |  |
| **Heel to toe stand** |  |  |  |  |  |  |  |  |  |  |  |  |  |  |  |  |
| Time (seconds)* | 8 | 9(3,10) | 5 | 11(8,15) | 6 | 13(10,30) | 6 | 13(10,30) | 3 | 15(5,30) | 3 | 11(9,15) | - | - | - | - |
| Sets* | 8 | 1(1,2) | 5 | 2(1,3) | 6 | 2(1,3) | 6 | 2(1,2) | 3 | 2(1,4) | 3 | 3(2,3) | - | - | - | - |
| Given as home exercise | 6 | 26.1% | 5 | 31.3% | 6 | 33.3% | 4 | 33.3% | 2 | 20.0% | 5 | 62.5% | 1 | 50.0% | 0 | 0.0% |
| **Heel to toe walking** |  |  |  |  |  |  |  |  |  |  |  |  |  |  |  |  |
| Reps* | 5 | 9(6,10) | 4 | 8(5,10) | 3 | 11(10,12) | 6 | 11(8,20) | 4 | 10(5,15) | 2 | 5(5,5) | 1 | 5(5,5) | 2 | 10(5,15) |
| Sets* | 5 | 2(1,2) | 4 | 2(1,3) | 3 | 1(1,2) | 6 | 2(1,3) | 4 | 2(1,2) | 2 | 2(2,2) | 1 | 2(2,2) | 2 | 3(1,5) |
| Given as home exercise | 4 | 17.4% | 4 | 25.0% | 2 | 11.1% | 5 | 41.7% | 5 | 50.0% | 4 | 50.0% | 2 | 100.0% | 2 | 66.7% |
| **Single leg stand** |  |  |  |  |  |  |  |  |  |  |  |  |  |  |  |  |
| Time (seconds)* | 7 | 8(3,10) | 2 | 12(8,15) | 7 | 12(5,25) | 4 | 12(8,20) | 4 | 11(8,15) | 2 | 16(12,20) | - | - | 1 | 10(10,10) |
| Sets* | 7 | 1(1,3) | 2 | 2(1,3) | 7 | 2(1,3) | 4 | 2(1,2) | 4 | 2(1,3) | 2 | 3(2,3) | - | - | 1 | 1(1,1) |
| Given as home exercise | 5 | 21.7% | 2 | 12.5% | 5 | 27.8% | 3 | 25.0% | 3 | 30.0% | 4 | 50.0% | 1 | 50.0% | 0 | 0.0% |
| **Heel walking** |  |  |  |  |  |  |  |  |  |  |  |  |  |  |  |  |
| Reps* | 2 | 7(3,10) | 2 | 13(6,20) | 1 | 10(10,10) | 2 | 10(10,10) | 1 | 10(10,10) | 1 | 5(5,5) | - | - | - | - |
| Sets* | 2 | 1(1,1) | 2 | 2(1,3) | 1 | 2(2,2) | 2 | 1(1,1) | 1 | 2(2,2) | 1 | 3(3,3) | - | - | - | - |
| Given as home exercise | 1 | 4.3% | 2 | 12.5% | 1 | 5.6% | 0 | 0.0% | 1 | 10.0% | 2 | 25.0% | 0 | 0.0% | 0 | 0.0% |
| **Toe walking** |  |  |  |  |  |  |  |  |  |  |  |  |  |  |  |  |
| Reps* | 2 | 7(3,10) | 2 | 8(6,10) | 1 | 10(10,10) | 2 | 10(10,10) | 2 | 12(8,15) | - | - | - | - | 1 | 5(5,5) |
| Sets* | 2 | 1(1,1) | 2 | 1(1,1) | 1 | 1(1,1) | 2 | 2(1,2) | 2 | 3(2,3) | - | - | - | - | 1 | 5(,55) |
| Given as home exercise | 2 | 8.7% | 2 | 12.5% | 2 | 11.1% | 1 | 8.3% | 2 | 20.0% | 1 | 12.5% | 0 | 0.0% | 1 | 33.3% |
| **Other treatment/s given** |  |  |  |  |  |  |  |  |  |  |  |  |  |  |  |  |
| No | 17 | 73.9% | 14 | 87.5% | 15 | 83.3% | 11 | 91.7% | 9 | 90.0% | 6 | 75.0% | 2 | 100.0% | 2 | 66.7% |
| Yes | 6 | 26.1% | 2 | 12.5% | 3 | 16.7% | 1 | 8.3% | 1 | 10.0% | 2 | 25.0% | 0 | 0.0% | 1 | 33.3% |
| **Supervised walk completed** |  |  |  |  |  |  |  |  |  |  |  |  |  |  |  |  |
| Yes | 17 | 73.9% | 12 | 75.0% | 15 | 83.3% | 9 | 75.0% | 8 | 80.0% | 5 | 62.5% | 0 | 0.0% | 2 | 66.7% |
| **Total walking time (minutes)** | 6 | 6(2,10) | 4 | 5(4,7) | 3 | 6(5,8) | 3 | 11(5,20) | 2 | 4(2,5) | 3 | 13(10,20) | 2 | 10(10,10) | 1 | 10(10,10) |
| Walking aid used | 2 | 8.7% | 2 | 12.5% | 2 | 11.1% | 2 | 16.7% | 0 | 0.0% | 1 | 12.5% | 0 | 0.0% | 0 | 0.0% |
| Walking stick | 2 | 8.7% | 1 | 6.2% | 2 | 11.1% | 2 | 16.7% | 0 | 0.0% | 1 | 12.5% | 0 | 0.0% | 0 | 0.0% |
| Crutches | 0 | 0.0% | 0 | 0.0% | 0 | 0.0% | 0 | 0.0% | 0 | 0.0% | 0 | 0.0% | 0 | 0.0% | 0 | 0.0% |
| 3-wheeled walker | 0 | 0.0% | 1 | 6.2% | 0 | 0.0% | 0 | 0.0% | 0 | 0.0% | 0 | 0.0% | 0 | 0.0% | 0 | 0.0% |
| 4-wheeled walker | 0 | 0.0% | 0 | 0.0% | 0 | 0.0% | 0 | 0.0% | 0 | 0.0% | 0 | 0.0% | 0 | 0.0% | 0 | 0.0% |
| **Walking challenges** |  |  |  |  |  |  |  |  |  |  |  |  |  |  |  |  |
| No challenges used | 4 | 17.4% | 2 | 12.5% | 1 | 5.6% | 1 | 8.3% | 0 | 0.0% | 1 | 12.5% | 0 | 0.0% | 0 | 0.0% |
| Steps / stairs | 2 | 8.7% | 1 | 6.2% | 1 | 5.6% | 1 | 8.3% | 2 | 20.0% | 2 | 25.0% | 2 | 100.0% | 1 | 33.3% |
| Ramp | 0 | 0.0% | 0 | 0.0% | 1 | 5.6% | 1 | 8.3% | 0 | 0.0% | 0 | 0.0% | 0 | 0.0% | 0 | 0.0% |
| Uneven surfaces | 0 | 0.0% | 0 | 0.0% | 1 | 5.6% | 1 | 8.3% | 0 | 0.0% | 0 | 0.0% | 1 | 50.0% | 1 | 33.3% |
| Carrying object | 0 | 0.0% | 0 | 0.0% | 1 | 5.6% | 1 | 8.3% | 0 | 0.0% | 1 | 12.5% | 1 | 50.0% | 0 | 0.0% |
| Increased speed | 0 | 0.0% | 0 | 0.0% | 0 | 0.0% | 0 | 0.0% | 0 | 0.0% | 1 | 12.5% | 1 | 50.0% | 1 | 33.3% |
| Other | 0 | 0.0% | 1 | 6.2% | 0 | 0.0% | 0 | 0.0% | 1 | 10.0% | 0 | 0.0% | 0 | 0.0% | 1 | 33.3% |
| **Appointment duration (minutes)*** | 24 | 52(0,60) | 21 | 23(0,45) | 21 | 24(0,35) | 16 | 21(0,30) | 11 | 26(0,30) | 8 | 31(30,35) | 3 | 21(0,33) | 3 | 23(15,30) |

* Exercise repetitions (reps), sets and weight used in exercises are presented as mean and range (minimum, maximum).
