# Supplementary material for: Tailored exercise management versus usual care for people aged 80 years or older with hip/knee osteoarthritis and comorbidities (TEMPO): multicentre feasibility randomised controlled trial in England
Source: BMJ Open. 2025 Sep 22;15(9):e104813. doi: 10.1136/bmjopen-2025-104813 (PMC12458626; doi:10.1136/bmjopen-2025-104813)
Supplement: online supplemental file 5 [file bmjopen-15-9-s005.docx]

**Supplementary Table S5. Summary of Home Exercise Diary in the TEMPO intervention**

|  | Week 1 | Week 2 | Week 3 | Week 4 | Week 5 | Week 6 | Week 7 | Week 8 | Week 9 | Week 10 | Week 11 | Week 12 |
| --- | --- | --- | --- | --- | --- | --- | --- | --- | --- | --- | --- | --- |
| Diary Completion**  n (%) | 18  (78.3%) | 17  (73.9%) | 17  (73.9%) | 16  (69.6%) | 16  (69.6%) | 15  (65.2%) | 15  (65.2%) | 13  (56.5%) | 12  (52.2%) | 8  (34.8%) | 8  (34.8%) | 6  (26.1%) |
| Number of days participants reported exercises were performed  n, median (IQR), (min, max) | 17,  5 (4,6), (3,7) | 16,  5 (4,5), (2,7) | 17,  5 (4,6), (3,7) | 16,  5 (4,6), (3,7) | 15,  5 (4,6), (1,7) | 13,  5 (5,5), (2,7) | 11,  5 (3,5), (1,7) | 11,  5 (5,5), (4,7) | 11,  5 (4,5), (4,7) | 8,  5 (5,6), (4,7) | 6,  4 (3,7), (2,7) | 5,  5 (4,6), (4,7) |
| Number of participants reporting no exercises perfomed | 1 | 1 | 0 | 1 | 1 | 2 | 4 | 2 | 1 | 1 | 2 | 1 |
| Number of days participants reported walk was performed  n, median (IQR), (min, max) | 13,  3 (3,6), (1,7) | 12,  5 (3,6), (2,7) | 14,  4 (2,5), (1,7) | 14,  5 (3,6), (1,7) | 14,  4 (2,7), (1,7) | 10,  5 (4,7), (1,7) | 8,  5 (4,7), (2,7) | 9,  5 (4,7), (1,7) | 9,  5 (3,6), (2,7) | 6,  5 (4,7), (2,7) | 6,  4 (2,7), (1,7) | 5,  4 (4,7), (2,7) |
| Number of participants reporting no walk was perfomed | 5 | 5 | 3 | 3 | 2 | 5 | 7 | 4 | 3 | 3 | 2 | 1 |
| Walk time per day (minutes)  n, mean (SD) | 13,  26.9 (17.5) | 12,  25.2 (10.5) | 13,  27.2 (18.03) | 13,  27.1 (16.3) | 13,  27.9 (18.6) | 10,  27.7 (17.2) | 7,  25.0 (17.7) | 9,  25.8 (13.3) | 9,  21.2 (9.3) | 6,  25.1 (3.7) | 6,  29.1 (10.01) | 5,  26.9 (9.6) |

* Expected number of paticipants completing diaries in each week is those who received the allocated intervention (n=23)

** Numbers presented are participants providing at least one diary in the specified week.
