# Supplementary material for: Tailored exercise management versus usual care for people aged 80 years or older with hip/knee osteoarthritis and comorbidities (TEMPO): multicentre feasibility randomised controlled trial in England
Source: BMJ Open. 2025 Sep 22;15(9):e104813. doi: 10.1136/bmjopen-2025-104813 (PMC12458626; doi:10.1136/bmjopen-2025-104813)
Supplement: online supplemental file 6 [file bmjopen-15-9-s006.docx]

**Supplementary Table S6. Usual care delivery**

|  | **Appointment**  **1** | | **Appointment 2** | | **Appointment 3** | | **Appointment 4** | | **Appointment 5** | | **Appointment 6** | | **Appointment 7** | | **Appointment 8** | |
| --- | --- | --- | --- | --- | --- | --- | --- | --- | --- | --- | --- | --- | --- | --- | --- | --- |
|  | **N = 23** | | **N = 18** | | **N = 9** | | **N = 2** | | **N = 0** | | **N = 0** | | **N = 0** | | **N = 0** | |
| **Mode of delivery** |  |  |  |  |  |  |  |  |  |  |  |  |  |  |  |  |
| In-person | 22 | 95.7% | 16 | 88.9% | 8 | 88.9% | 2 | 100% | 0 | 0.0% | 0 | 0.0% | 0 | 0.0% | 0 | 0.0% |
| via Telephone | 1 | 4.3% | 2 | 11.1% | 1 | 11.1% | 0 | 0.0% | 0 | 0.0% | 0 | 0.0% | 0 | 0.0% | 0 | 0.0% |
| Provision of walking aid | 2 | 7.7% | 0 | 0.0% | 1 | 3.8% | 0 | 0.0% | 0 | 0.0% | 0 | 0.0% | 0 | 0.0% | 0 | 0.0% |
| Provision of information booklet (paper or electronic) | 6 | 23.1% | 2 | 7.7% | 0 | 0.0% | 0 | 0.0% | 0 | 0.0% | 0 | 0.0% | 0 | 0.0% | 0 | 0.0% |
| Electrotherapy | 0 | 0.0% | 0 | 0.0% | 0 | 0.0% | 0 | 0.0% | 0 | 0.0% | 0 | 0.0% | 0 | 0.0% | 0 | 0.0% |
| Manual therapy | 0 | 0.0% | 0 | 0.0% | 0 | 0.0% | 0 | 0.0% | 0 | 0.0% | 0 | 0.0% | 0 | 0.0% | 0 | 0.0% |
| Exercises | 18 | 69.2% | 13 | 50% | 7 | 26.9% | 2 | 7.7% | 0 | 0.0% | 0 | 0.0% | 0 | 0.0% | 0 | 0.0% |
| Home exercise program | 22 | 84.6% | 18 | 69.2% | 10 | 38.5% | 2 | 7.7% | 0 | 0.0% | 0 | 0.0% | 0 | 0.0% | 0 | 0.0% |
| Referral to other health professional^2^ | 0 | 0.0% | 0 | 0.0% | 1 | 3.8% | 0 | 0.0% | 0 | 0.0% | 0 | 0.0% | 0 | 0.0% | 0 | 0.0% |
| Other treatment(s)^3^ | 6 | 23.1% | 2 | 7.7% | 2 | 7.7% | 0 | 0.0% | 0 | 0.0% | 0 | 0.0% | 0 | 0.0% | 0 | 0.0% |

^1^ The denominator represents the total number of participants attending each session 26.

^2^ Referral (n=1): Enhanced role physiotherapist.

^3^ Other treatment: Advice, Education, Gait education, Advice about exercise, functional actions and treatment plan, Advice on OA management, Patient cancelled due to fall and fracture
