# Supplementary material for: Tailored exercise management versus usual care for people aged 80 years or older with hip/knee osteoarthritis and comorbidities (TEMPO): multicentre feasibility randomised controlled trial in England
Source: BMJ Open. 2025 Sep 22;15(9):e104813. doi: 10.1136/bmjopen-2025-104813 (PMC12458626; doi:10.1136/bmjopen-2025-104813)
Supplement: Supplementary file 7 [file bmjopen-2025-104813.reviewer_comments.pdf]

PEER REVIEW HISTORY

BMJ Open publishes all reviews undertaken for accepted manuscripts. Reviewers are asked to complete a checklist review form and are provided with free text boxes to elaborate on their assessment. These free text comments are reproduced below.

ARTICLE DETAILS

Title (Provisional)

Tailored exercise management versus usual care for people aged 80 years or older with hip/knee osteoarthritis and comorbidities (TEMPO): multicentre feasibility randomised controlled trial in England.

Authors

Nicolson, Philippa JA; Holden, Melanie A; Marian, Ioana; Saeedi, Elnaz; Williamson, Esther; Moylan, Dianna; Stone, Megan; Hopewell, Sally; Lamb, Sarah Elizabeth; feasibility trial collaborators, TEMPO

VERSION 1 - REVIEW

**Reviewer**	**1**
**Name**	**Moezy, Azar**
**Affiliation Department**	**Hazrat-e Rasool General Hospital, Sports Medicine**
**Date**	**04-Jun-2025**
**COI**	**None**

Thank you for the opportunity to review the paper entitled “Tailored exercise management versus usual care for people

aged 80 years or older with hip/knee osteoarthritis and

comorbidities (TEMPO): multicentre randomised controlled

feasibility trial.”

For the authors

- The study is well-designed, with clearly outlined feasibility objectives and thoughtful inclusion of patient and public involvement. The writing is clear and the findings are of interest to clinicians and researchers working with older populations.
- Only 52% of participants received the minimum 4 sessions of the tailored programme, placing it in the "amber" category. While reasons for missed sessions are

discussed, more emphasis should be placed on how these barriers (e.g. scheduling capacity, participant illness) will be addressed in a future definitive trial.

- With 96.1% of participants identifying as White British, the sample lacks representativeness. The discussion acknowledges this, but concrete plans for increasing diversity (e.g. translated materials, community engagement strategies) could be expanded upon.
- The NEADL scale demonstrated a significant ceiling effect. It may not be sufficiently sensitive to detect meaningful change in this cohort. Consider suggesting alternative or supplementary outcome measures for future trials that can better capture functional variation in older adults.
- There appears to be some overlap in care received by both arms, particularly for participants referred to NHS physiotherapy in the usual care group. This may confound group differences. The authors may consider strengthening the comparator definition in the main trial protocol.
- While the manuscript is generally well-written, some sections—particularly the methods and intervention descriptions—are dense and may benefit from minor editorial tightening for improved readability.
- Consider including brief demographic comparisons between participants who completed vs. did not complete all intervention sessions, if available.
- The discussion might benefit from acknowledging the implications of digital delivery (e.g. video consultations) for participants in this age group.

**Reviewer**	**2**
**Name**	**Latimer, Lorna**
**Affiliation**	**NIHR Leicester Biomedical Research Centre**
**Date**	**24-Jun-2025**
**COI**	**None**

The authors should be congratulated on successfully recruiting their target number of participants for a feasibility RCT within their target time period and for collecting outcome measures in a high proportion of those participants at baseline and follow-up.

To summarise my opinion of the manuscript, I feel that clarification regarding the rationale for the choice of study population (with relation to comorbidities) is required. Importantly the interpretation of the results should be considered with consideration of a suitable declared primary outcome measure and reference should be made to a power calculation to establish required sample size before an assessment of feasibility for a future trial can be declared. It is encouraging for the authors that four sites were able to recruit on average 1.5 participants per month for an RCT of an exercise-based intervention.

I have set out some general and specific points below. Thank you for the opportunity to review this interesting work.

In the Introduction section, please could the authors set out why they targeted a population with hip / knee OA plus at least one comorbidity? What proportion of the hip / knee OA population does this represent? Is it the majority of patients, or is there a significant proportion of the hip / knee OA population who do not have any comorbidities and were therefore excluded from the trial?

Participants and outcome assessors were not blinded to treatment allocation. It is entirely understandable that the participants were not blinded, however, having blinded outcome assessors would significantly reduce the risk of bias in an RCT. Please could the authors comment on whether they think is is possible to have blinded outcome assessors in the full RCT and what implications this might have for the delivery of the trial.

49/51 participants were white British. The authors acknowledge this as a significant limitation. Did the population recruited reflect local demographics? Were any specific efforts made to reach non-white groups in this trial?

The protocol paper for this trial stated an aim of estimating a sample size for “definitive” RCT. Please could the authors comment on their plans for sample size calculation using data from this feasibiltiy trial with reference to a primary outcome?

There are repeated references in this paper to testing the feasibility of a future “definitive trial”. What do the authors mean by this term? Can any single trial provide evidence to determine treatment options for all patients with knee / hip OA and one or more comorbid conditions?

Specific comments:

Abstract – 133 screened, 94 eigible, so 39 should have been ineligible. Paper reports 43 were ineligible.

Line 71, 16 RCTs investigating the effects of exercise therapy in people with OA over the age of 80 sounds like quite a lot. Please can the authors expand on why these 16 trials do not give sufficient evidence and another “definitive trial” is required, and what it will do differently to these previous trials.

Page 9, Inclusion / exclusion criteria. Please could a full bulleted list of inclusion and exclusion criteria be added to the supplement with a narrative summary in the main body of the paper.

Line 150, Blinding: “The local researcher clinicians conducting the baseline and 14-week assessments were independent of the clinical team.” Does this mean that they were blinded? Please clarify, as it seems to contradict what is stated in the abstract relating to blinding.

How does the TEMPO intervention differ to usual care in the practices used for recruitment?

Supplementary Table S1. In the “How” line please clarify whether appointments 5-8 were optionally face-to-face in clinic (in-person assessments), or a one-on-one “face-to-face” (remote). Or were they all remote?

Given the intervention included elements to “challenge balance” during walking, were there any adverse events (that did not meet the threshold of a serious adverse event)?

Line 268, Sample size: “The target sample size was a minimum of 50 participants in at least four sites over a maximum of 9 months." Why? What is the justification for this sample size and recruitment rate? Does this arise from a power calculation to determine the sample size required for a future, similarly designed trial with a primary outcome measure that was also collected in this feasibility trial?

Line 272 onwards, Statistical analysis: outcome data were analysed on an ITT basis using linear regression. The authors state that no p values are presented due to lack of power. As this is a feasibility study, it would be more appropriate to present descriptive statistics only and focus on the number of outcome measures successfully performed at each time point. Outcome data themselves are not relevant, unless to be used for powering a future trial.

Line 310, Feasibility Results: please give a description of the results rather than just referring to the figure. This is the primary outcome of the study.

Flow of participants figure. Were any potential participants ineligible because they had zero comorbidities? Do you know what proportion of the knee/hip OA population have no comorbidities? Is there a reason to exclude people without comorbidities from the trial?

Feasibility criteria: criteria are clearly stated to define feasibility based on number of participants identified, consented, completed and who adhered to the intervention. What is the basis for these criteria? What is the anticipated primary outcome for the “future

definitive trial”? Do the authors have the data they require to perform a power calculation informing the sample size required for a future trial, and what design would it be: superiority, non-inferiority etc?

Was any qualitative work conducted with participants to assess appeal and acceptability of the intervention to decliners / accepters? Or with healthcare professionals to gauge possible reasons for low uptake of the intervention in non-white groups?

Line 392, Discussion: Given that the design of the design of the “definitive trial” of an exercise training intervention in hip / knee OA patients over the age of 80 years is not clearly stated in the manuscript, the statement “Our feasibility RCT demonstrated that a definitive trial comparing a 12-week tailored exercise programme versus usual care for people aged 80 years or older with hip/knee osteoarthritis and comorbidities is feasible” is justified. The primary outcome of such a trial is mentioned in the results section, but the authors highlight significant problems with the proposed outcome measure (NEADL) in this population with a ceiling effect apparent. The team behind the present study should be congratilated for successfully recruiting people with hip / knee OA to their feasibilty RCT and completing a range of outcome measures, but I would prefer a more cautious and precise appraisal of the implications of the results.

The authors describe limited capacity within the services delivering the intervention as a reason why only around half of the intervention group received four or more sessions and suggest ensuring protected time for clinicians or extension of the intervention period to improve programme completion. On this point I would urge the authors to consider what the question is that they would try to answer in a future trial: does a perfectly delivered intervention work in this population; or within the realistic limitations of a given healthcare setting, does this intervention work in the real world?

Line 460: I think this is the first mention of cost effectiveness. I don’t believe any health economic data are presented in the results. Were these data successfully collected?

Flow of participants figure: n=3 in usual care group did not receive the allocated intervention. Please could you explain?

Minor comments:

Line 172: missing word? “which the <<participant?>> filled out”

VERSION 1 - AUTHOR RESPONSE

Reviewer: 1
Dr. Azar Moezy, Hazrat-e Rasool General Hospital

Comments to the Author:
Thank you for the opportunity to review the paper entitled “Tailored exercise

management versus usual care for people aged 80 years or older with hip/knee osteoarthritis and comorbidities (TEMPO): multicentre randomised controlled feasibility trial."
The study is well-designed, with clearly outlined feasibility objectives and thoughtful inclusion of patient and public involvement. The writing is clear, and the findings are of interest to clinicians and researchers working with older populations.

Only 52% of participants received the minimum 4 sessions of the tailored programme, placing it in the "amber" category. While reasons for missed sessions are discussed, more emphasis should be placed on how these barriers (e.g. scheduling capacity, participant illness) will be addressed in a future definitive trial.
Lines 451-453 of the Discussion proposes potential changes to overcome these barriers: *"A future RCT should ensure that clinician's time is protected for the delivery of trial intervention sessions or consider the intervention period be extended to 16 weeks."*
As detailed in Lines 494-496 of the Discussion, we have formed a Community of Practice with patients, clinicians and researchers to discuss and decide on strategies to overcome barriers to intervention delivery and improve the representativeness of participants recruited to a future main trial. The Community of Practice will meet five times over the remainder of this year.
Concrete plans for changes to trial methods will be detailed in a future main trial protocol paper. No change made to manuscript.

With 96.1% of participants identifying as White British, the sample lacks representativeness. The discussion acknowledges this, but concrete plans for increasing diversity (e.g. translated materials, community engagement strategies) could be expanded upon.
We have undertaken community engagement meetings with three different underserved communities in England, and as outlined above, have formed a Community of Practice to discuss and decide on strategies to overcome barriers to intervention delivery and improve the representativeness of participants recruited to a future main trial.
Lines 428-431 outline potential changes, and concrete plans will be detailed in a future main trial protocol paper.
"*Potential modifications in a future RCT may include alternative methods of recruitment such as advertising in community settings, visiting community groups to speak about the trial and to answer questions, and producing easy read and/or translated versions of the patient information sheet."*

The NEADL scale demonstrated a significant ceiling effect. It may not be sufficiently sensitive to detect meaningful change in this cohort. Consider suggesting alternative or supplementary outcome measures for future trials that can better capture functional variation in older adults.
Addition made to Discussion, Lines 441-445: "*Primary outcome selection for a definitive future main trial should ensure that a wide variety of abilities can be assessed, and changes over time can be accurately captured for all participants. Utilising co-primary outcomes of the SPPB and EQ-5D-5L would enable objective assessment of functional change, and broader self-reported assessment of health-related quality of life."*

There appears to be some overlap in care received by both arms, particularly for participants referred to NHS physiotherapy in the usual care group. This may confound group differences. The authors may consider strengthening the comparator definition in the main trial protocol.
Thank you for this suggestion, we acknowledge that there certainly was overlap between the intervention and usual care treatments. We chose not to define Usual Care for this feasibility trial to gain insight into what was delivered by clinicians. If we proceed to a main trial we plan to define Usual Care, and this will be determined following our process evaluation and Community of Practice which is currently underway.

While the manuscript is generally well-written, some sections—particularly the methods and intervention descriptions—are dense and may benefit from minor editorial tightening for improved readability.
We have edited the methods to improve readability, while keeping the level of detail required so readers do not have to locate and read the protocol paper to understand the trial methods fully. Deleted text is shown with strikethrough and highlighted in the Marked Manuscript; we have not included the deleted text in our response here as there are multiple edits.

Consider including brief demographic comparisons between participants who completed vs. did not complete all intervention sessions, if available.
Summary text added to Results, Lines 354-357: *"When compared to compared to those who attended four or more sessions, participants who attended less than four sessions were more likely to be male (42% vs 15%), less likely to live alone (50% vs 69%), or use a walking aid inside (25% vs 46%) and reported being more confident walking ½ mile (median score: 8.5/10 vs 3.0)."*

The discussion might benefit from acknowledging the implications of digital delivery (e.g. video consultations) for participants in this age group.
Text added to Discussion, Lines 457-463: *"The protocol included the option for TEMPO sessions 5-8 to be delivered via telephone or videocall at the discretion of the participant and physiotherapist. There was very little uptake of remote delivery (3 sessions were delivered by telephone across the TEMPO group, and 0 by videocall). Future trial development should give consideration to overcoming barriers to uptake of video consultations in this population group, as they offer an alternative to overcome transport and hospital access difficulties."*

Reviewer: 2
Dr. Lorna Latimer, NIHR Leicester Biomedical Research Centre, University of Leicester

Comments to the Author:
The authors should be congratulated on successfully recruiting their target number of participants for a feasibility RCT within their target time period and for collecting outcome measures in a high proportion of those participants at baseline and follow-up.
To summarise my opinion of the manuscript, I feel that clarification regarding the rationale for the choice of study population (with relation to comorbidities) is required. Importantly

the interpretation of the results should be considered with consideration of a suitable declared primary outcome measure and reference should be made to a power calculation to establish required sample size before an assessment of feasibility for a future trial can be declared. It is encouraging for the authors that four sites were able to recruit on average 1.5 participants per month for an RCT of an exercise-based intervention.
I have set out some general and specific points below. Thank you for the opportunity to review this interesting work.

In the Introduction section, please could the authors set out why they targeted a population with hip / knee OA plus at least one comorbidity? What proportion of the hip / knee OA population does this represent? Is it the majority of patients, or is there a significant proportion of the hip / knee OA population who do not have any comorbidities and were therefore excluded from the trial?
Text added to Introduction, Lines 63-66 to clarify: "*Hip and/or knee OA has one of the highest rates of comorbidity (68% to 85%) among all chronic conditions (6-8). In a UK general practice population of adults aged 50 years and older people with OA had 2.35 times higher risk of having comorbidities compared to people without OA (9). The presence of comorbidities is also increasingly common with age, meaning people aged 80 years and older with hip and/or knee OA are most likely to also be managing at least one other chronic health condition.*"

Addition also made to Lines 70-74: "*Our previous research, and that of others, has found that comorbidity increases the likelihood of poor physical function in patients with OA, and the combined influence is greater than would be expected from the influence of either OA or the comorbid conditions alone (9, 13). Consequently, addressing comorbidities is crucial in optimising management of OA (9).*"

Participants and outcome assessors were not blinded to treatment allocation. It is entirely understandable that the participants were not blinded, however, having blinded outcome assessors would significantly reduce the risk of bias in an RCT. Please could the authors comment on whether they think is possible to have blinded outcome assessors in the full RCT and what implications this might have for the delivery of the trial.
Yes, we plan to have blinded outcome assessors in the main trial, by having randomisation and patient notification of group allocation completed by the central trial team rather than at participating sites.
Within the financial constraints of a feasibility trial being run within a fellowship the Chief Investigator also fulfilled the roles of trial manager and trial physiotherapist (while working 0.6FTE). As such it was not possible to centralise undertaking randomisation and notifying patients of their group allocation.

49/51 participants were white British. The authors acknowledge this as a significant limitation. Did the population recruited reflect local demographics? Were any specific efforts made to reach non-white groups in this trial?
The non-representative nature of the recruited cohort is certainly a limitation, which we are working to address through our current Community Engagement meetings and Community of Practice, to ensure this is overcome in a future main trial.

As detailed in the trial protocol, we purposively recruited sites that represented a range of geographical and clinical settings, and who typically saw patients from a range of socio-demographic backgrounds. We did this by examining IMD profiles for each potential site, and having local PI's submit summary statistics of patients who would potentially be eligible for the trial, before we decided to proceed with them as a trial site. We did not make specific efforts to reach non-white groups in this feasibility trial, but as detailed in the discussion and our responses to other comments we plan to do so in the main trial.

The protocol paper for this trial stated an aim of estimating a sample size for "definitive" RCT. Please could the authors comment on their plans for sample size calculation using data from this feasibility trial with reference to a primary outcome?

Given the observed limitations of the proposed primary outcome of the NEADL we have given significant thought to what should be the primary outcome if we proceed to a main trial. One approach we are considering is to use co-primary outcomes of the SPPB and the EQ-5D-5L.

Text added to Discussion, Lines 441-445 in response to a comment from Reviewer 1:

"*Primary outcome selection for a definitive future main trial should ensure that a wide variety of abilities can be assessed, and changes over time can be accurately captured for all participants. Utilising co-primary outcomes of the SPPB and EQ-5D-5L would enable objective assessment of functional change, and broader self-reported assessment of health-related quality of life."*

There are repeated references in this paper to testing the feasibility of a future "definitive trial". What do the authors mean by this term? Can any single trial provide evidence to determine treatment options for all patients with knee / hip OA and one or more comorbid conditions?

We agree that no single trial can provide evidence to determine treatment options for all patients with knee/hip OA and comorbidities. However, the use of the term 'definitive trial' is widely used in randomised controlled trial guidance and literature, particularly in the context of differentiating from a feasibility or pilot trial. A definitive randomised controlled trial is defined as a large-scale, robust study designed to assess the effectiveness or efficacy of an intervention, treatment, or therapy. It is considered the gold standard for evaluating interventions.

(Eldridge SM, Lancaster GA, Campbell MJ, Thabane L, Hopewell S, Coleman CL, Bond CM. Defining Feasibility and Pilot Studies in Preparation for Randomised Controlled Trials: Development of a Conceptual Framework. PLoS One. 2016 Mar 15;11(3):e0150205.)

In accordance with this guidance our protocol and objectives used the term definitive trial, so we have used the term throughout the manuscript to be consistent.

Specific comments:

Abstract – 133 screened, 94 eigible, so 39 should have been ineligible. Paper reports 43 were ineligible.

Text corrected, Lines 19-20: *"The main reasons for ineligibility were symptoms not consistent with hip or knee osteoarthritis (10/39; 25.6%) or already having had a physiotherapy appointment (8/39; 20.5%)"*.

Line 71, 16 RCTs investigating the effects of exercise therapy in people with OA over the age of 80 sounds like quite a lot. Please can the authors expand on why these 16 trials do not give sufficient evidence and another "definitive trial" is required, and what it will do differently to these previous trials.

The systematic review included exercise trials in any population over 80, not just those with osteoarthritis. None of the included trials were specifically populations with osteoarthritis, and only one trial in people 80 and over with osteoarthritis has been published since.

The sentence the reviewer refers to reads in full:

"Our systematic review of the effects of therapeutic exercise on physical and psychosocial outcomes in any community-dwelling adults aged 80 years or older identified only 16 published RCTs (16)"

Further, Lines 79-81 state*: "Of the 96 RCTs included in the recently published Cochrane review of exercise for knee OA (14), only one trial recruited participants with an average age over 80 years (15)."*

No change made to manuscript.

Page 9, Inclusion / exclusion criteria. Please could a full bulleted list of inclusion and exclusion criteria be added to the supplement with a narrative summary in the main body of the paper.

Text edited, Lines 138-146 and Supplementary Table S1 added: *"Full inclusion and exclusion criteria are detailed in Supplementary Table S1. The target population was adults aged 80 years or older with clinical hip and/or knee OA (hereafter referred to as hip/knee OA) and one or more comorbidity.*

Participants were excluded if they had a terminal illness, significant cognitive impairment, were unable to walk 3 metres with or without an aid or were unable to follow verbal or written instructions."

Line 150, Blinding: "The local researcher clinicians conducting the baseline and 14-week assessments were independent of the clinical team." Does this mean that they were blinded? Please clarify, as it seems to contradict what is stated in the abstract relating to blinding.

Local researcher clinicians were independent of the clinical team who delivered the TEMPO intervention. They were not blinded to group allocation as they completed randomisation following baseline assessment and informed the participant of their group allocation.

Text added to Lines 165-167 to clarify:

"The local researcher clinicians conducting the baseline and 14-week assessments were independent of the clinical team who delivered the tailored exercise programme."

How does the TEMPO intervention differ to usual care in the practices used for recruitment?

We chose not to define Usual Care for this feasibility trial to gain insight into this question. Lines 465-469 in the Discussion *"We did not define the content of usual care for this feasibility trial. Participants randomised to usual care received a median of two physiotherapy sessions and were most commonly prescribed exercises and a home exercise program. This level of input does not differ significantly from the care received in the intervention arm, and this would need to be considered for a future RCT."*

Supplementary Table S1. In the "How" line please clarify whether appointments 5-8 were optionally face-to-face in clinic (in-person assessments), or a one-on-one "face-to-face" (remote). Or were they all remote?
Lines 178-180 in the manuscript states *"Sessions five to eight were at the discretion of the physiotherapist and the participant, and could be delivered in-person, via video consultation or telephone call."*
Supplementary Table S2 (numbering updated following addition of another Supplementary table), 'How' row edited to clarify: "face to face" replaced by "In clinic"

Given the intervention included elements to "challenge balance" during walking, were there any adverse events (that did not meet the threshold of a serious adverse event)?
No there were no adverse events or near misses reported by clinicians within the exercise sessions. The treatment intervention logs included a space to record any near misses or adverse events for the research team.

Line 268, Sample size: "The target sample size was a minimum of 50 participants in at least four sites over a maximum of 9 months." Why? What is the justification for this sample size and recruitment rate? Does this arise from a power calculation to determine the sample size required for a future, similarly designed trial with a primary outcome measure that was also collected in this feasibility trial?
The main feasibility objective, and the basis of the sample size estimate was participant recruitment per centre. The sample size was based on a target of recruiting 1.5 participants per month per site
Text added to Methods, Lines 285-287: *"The sample size was considered sufficient to answer our feasibility objectives and assess the a priori progression criteria based on Teare et al recommendations (44)."*

Line 272 onwards, Statistical analysis: outcome data were analysed on an ITT basis using linear regression. The authors state that no p values are presented due to lack of power. As this is a feasibility study, it would be more appropriate to present descriptive statistics only and focus on the number of outcome measures successfully performed at each time point. Outcome data themselves are not relevant, unless to be used for powering a future trial.
We agree that the study was not powered to detect definitive treatment effects between groups and the outcome measures should be presented as descriptive statistics to primarily demonstrate the feasibility of collecting data.
In line with our pre-specified published protocol and the guidelines in the CONSORT extension for Pilot and Feasibility studies, we have additionally included between group differences together with confidence intervals to help assess potential signals of efficacy in outcome measures and provided the precision of estimates. We have emphasised that these comparisons are exploratory and hypothesis-generating only.

Line 310, Feasibility Results: please give a description of the results rather than just referring to the figure. This is the primary outcome of the study.
The 7 paragraphs following Line 310 describe the feasibility results (Lines 329-394 in Marked manuscript).

To avoid confusion, we have moved the sentence referring to the table to follow the text describing the results, rather than being before the text as it was previously.

Flow of participants figure. Were any potential participants ineligible because they had zero comorbidities? Do you know what proportion of the knee/hip OA population have no comorbidities? Is there a reason to exclude people without comorbidities from the trial?
As detailed in Figure 1 no potential participants were ineligible because they had no comorbidities. Detail has been added to the Introduction in response to the reviewer's earlier comment regarding comorbidities.
We chose to recruit people with comorbidities as previous exercise for OA trials often explicitly excluded these people.

Feasibility criteria: criteria are clearly stated to define feasibility based on number of participants identified, consented, completed and who adhered to the intervention. What is the basis for these criteria? What is the anticipated primary outcome for the "future definitive trial"? Do the authors have the data they require to perform a power calculation informing the sample size required for a future trial, and what design would it be: superiority, non-inferiority etc?
The basis for the feasibility criteria is outlined in Lines 228-230: *"The main uncertainty was whether enough participants would be able to be recruited and retained in the study until 14-week follow-up."*
Lines 252-253 state "The NEADL was the proposed primary outcome for a definitive trial."
In our response to the Reviewers earlier comment regarding the primary outcome for a future trial we detail the text we have added outlining one potential approach to use co-primary outcomes, both of which were included in the feasibility trial, meaning we have the data required to perform a power calculation.
Specifics of a future main trial are not yet decided. A hybrid implementation-effectiveness trial, using a stepped wedge approach is a design we are considering, to enable large-scale evaluation of both effectiveness of the intervention and the ability to implement it across multiple NHS sites.

Was any qualitative work conducted with participants to assess appeal and acceptability of the intervention to decliners / accepters? Or with healthcare professionals to gauge possible reasons for low uptake of the intervention in non-white groups?
Yes, as detailed in Lines 118-121 *"An embedded qualitative study including both patient participants and physiotherapists who delivered the intervention was also completed as part of a process evaluation which will be reported separately."*

Line 392, Discussion: Given that the design of the "definitive trial" of an exercise training intervention in hip / knee OA patients over the age of 80 years is not clearly stated in the manuscript, the statement "Our feasibility RCT demonstrated that a definitive trial comparing a 12-week tailored exercise programme versus usual care for people aged 80 years or older with hip/knee osteoarthritis and comorbidities is feasible" is justified. The primary outcome of such a trial is mentioned in the results section, but the authors highlight significant problems with the proposed outcome measure (NEADL) in this population with a ceiling effect apparent. The team behind the present study should be congratulated for successfully recruiting people with hip / knee OA to their feasibilty RCT

and completing a range of outcome measures, but I would prefer a more cautious and precise appraisal of the implications of the results.
The sentence the reviewer refers to reads in full: "*Our feasibility RCT demonstrated that a definitive trial comparing a 12-week tailored exercise programme versus usual care for people aged 80 years or older with hip/knee osteoarthritis and comorbidities is feasible, but modifications should be considered prior to a definitive trial."*
The authors feel this is an acceptably cautious and accurate appraisal of the implications of the results. As detailed in our response to the reviewer's earlier comment regarding feasibility criteria, it is not appropriate within this results manuscript for the feasibility trial to specify the exact methodology of a potential future trial, given that we are currently undertaking a process evaluation, community engagement and a community of practice to work with patients and clinicians to decide on these factors.

The authors describe limited capacity within the services delivering the intervention as a reason why only around half of the intervention group received four or more sessions and suggest ensuring protected time for clinicians or extension of the intervention period to improve programme completion. On this point I would urge the authors to consider what the question is that they would try to answer in a future trial: does a perfectly delivered intervention work in this population; or within the realistic limitations of a given healthcare setting, does this intervention work in the real world?
Thank you for this suggestion, this is certainly something we have given significant thought to and is a crucial point in whether an intervention is realistically going to be able to be implemented widely beyond a clinical trial setting. As detailed in response to earlier Reviewer comments, we are currently undertaking a process evaluation of the interventions and trial processes before finalising trial methods and proceeding to pursue funding for a main trial.
No change made to manuscript.

Line 460: I think this is the first mention of cost effectiveness. I don't believe any health economic data are presented in the results. Were these data successfully collected?
The EQ-5D-5L was collected as detailed in Lines 254-260. The EQ-5D is the NICE-recommended instrument for measuring health-related quality of life for cost-effectiveness analyses. As reported in Table 4 and Lines 349-350, the EQ-5D-5L was completed by 45/51 (88.2%) of participants.
No change made to manuscript.

Flow of participants figure: n=3 in usual care group did not receive the allocated intervention. Please could you explain?
One participant from the usual care group withdrew (Lines 344-347) and two were lost to follow-up, so no information was obtained on what treatment they received.

Minor comments:

Line 172: missing word? "which the <> filled out"
Line 172: "the" has been corrected to "they". The sentence now reads: *"to complete their home exercises and an exercise diary which they filled out and returned at each session."*

VERSION 2 - REVIEW

**Reviewer**	**2**
**Name**	**Latimer, Lorna**
**Affiliation**	**NIHR Leicester Biomedical Research Centre**
**Date**	**01-Aug-2025**
**COI**	

Thank you to the authors for addressing all of my comments, particularly for the clear explanation of the choice of population and relevance of comorbidities. I am satisfied with all of the responses with the exception of one minor point (detailed below). I hope to see this paper published in BMJ Open in the near future and that the authors are able to progress this work into a fully powered trial.

Line 164 (marked version of manuscript): "Physiotherapists delivering the tailored exercise programme and study participants were 165 told the treatment allocation. The local researcher clinicians conducting the baseline and 14-week assessments were independent of the clinical team who delivered the tailored exercise programme.". Although it it is stated clearly in the abstract, please make clear in this section that the outcome assessors were unblinded.

VERSION 2 - AUTHOR RESPONSE

Reviewer: 2
Dr. Lorna Latimer, NIHR Leicester Biomedical Research Centre, University of Leicester
Comments to the Author:
Thank you to the authors for addressing all of my comments, particularly for the clear explanation of the choice of population and relevance of comorbidities. I am satisfied with all of the responses with the exception of one minor point (detailed below). I hope to see this paper published in BMJ Open in the near future and that the authors are able to progress this work into a fully powered trial.
Thank you.

Line 164 (marked version of manuscript): "Physiotherapists delivering the tailored exercise programme and study participants were 165 told the treatment allocation. The local researcher clinicians conducting the baseline and 14-week assessments were independent of the clinical team who delivered the tailored exercise programme.". Although it it is stated clearly in the abstract, please make clear in this section that the outcome assessors were unblinded.

Lines 136-139 edited to clarify: *“Physiotherapists delivering the tailored exercise programme and study participants were told the treatment allocation. The local researcher clinicians conducting the baseline and 14-week assessments were independent of the clinical team who delivered the tailored exercise programme, and were not blinded to treatment allocation.”*
